# Surmounting Byproduct Inhibition in an Intermolecular Catalytic Asymmetric Alkene Bromoesterification Reaction as Revealed by Kinetic Profiling

**DOI:** 10.1021/acs.joc.3c00672

**Published:** 2023-06-16

**Authors:** D. Christopher Braddock, Ben M. J. Lancaster, Christopher J. Tighe, Andrew J. P. White

**Affiliations:** † Department of Chemistry, Molecular Sciences Research Hub, Imperial College London, White City Campus, 82 Wood Lane, London W12 0BZ, U.K.; ‡ Department of Chemical Engineering, Imperial College London, South Kensington Campus, Imperial College Road, London SW7 2AZ, U.K.

## Abstract

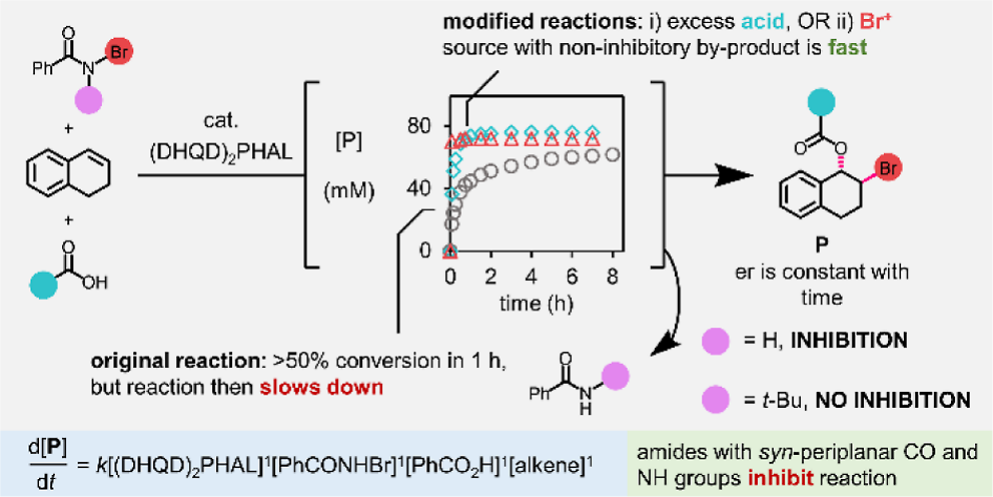

Kinetic profiling has shown that a (DHQD)_2_PHAL-catalyzed intermolecular asymmetric alkene bromoesterification reaction is inhibited by primary amides, imides, hydantoins, and secondary cyclic amides, which are byproducts of common stoichiometric bromenium ion sources. Two approaches to resolving the inhibition are presented, enabling the (DHQD)_2_PHAL loading to be dropped from 10 to 1 mol % while maintaining high bromoester conversions in 8 h or less. Iterative post-reaction recrystallizations enabled a homochiral bromonaphthoate ester to be synthesized using only 1 mol % (DHQD)_2_PHAL.

## Introduction

Alkene halofunctionalizations are powerful tools for the regiocontrolled and diastereoselective construction of carbon–heteroatom bonds.^1^ Since Borhan’s pioneering studies in 2010,^2^ many organocatalytic asymmetric *intramolecular* alkene bromofunctionalization reactions have been published, furnishing compounds such as bromolactones,^3^ cyclic bromoethers and bromoamines,^4^ and bromocarbocycles,^5^ in moderate to high er. On the other hand, the more entropically demanding *intermolecular* bromofunctionalization reactions of alkenes have been examined less thoroughly.^1c^ Some examples of catalytic asymmetric intermolecular alkene bromoesterification,^6^ bromoetherification,^7^ bromoamination,^8^ and bromohydroxylation^9^ of both functionalized and unfunctionalized alkenes have been sporadically reported in the past decades. However, these examples lack a kinetic study to investigate and improve a low catalytic turnover, low enantioselectivity, and long reaction times. Herein, kinetic profiling methods, time-adjusted analysis,^10^ and variable time normalization analysis^11^ have been used to explore an intermolecular alkene bromoesterification reaction catalyzed by (DHQD)_2_PHAL, originally reported by Shi *et al.* in 2014.^6a^ These experiments show that catalytic turnover is limited by inhibition mediated by the byproduct, which, when resolved (*vide infra*), allowed the reduction of catalyst loadings and reaction times. Furthermore, using a nominal 1 mol % loading of (DHQD)_2_PHAL, a homochiral bromoester could be obtained following a *post*-reaction recrystallization regime.

## Results and Discussion

**Figure 1:**
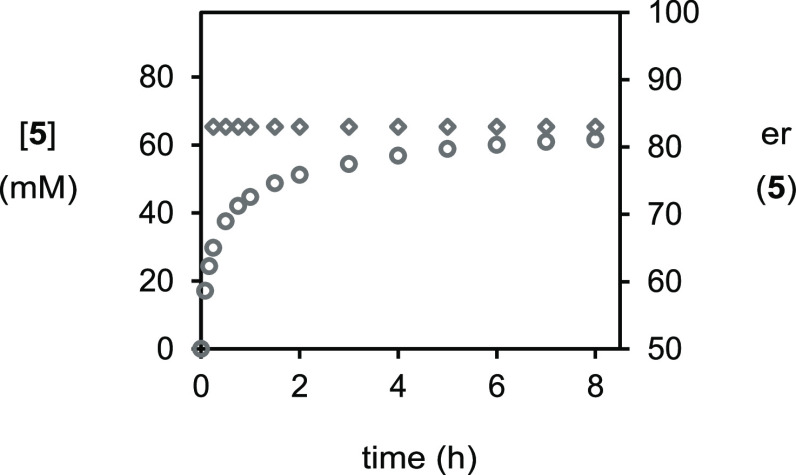
Plot of [**5**] (○) and er (◇) *vs* time as monitored by HPLC methods; [dialin]_0_ = 80 mM, [PhCO_2_H]_0_ = 96 mM, [PhCONHBr]_0_ = 96 mM, and [(DHQD)_2_PHAL]_0_ = 8 mM.

**Scheme 1:**
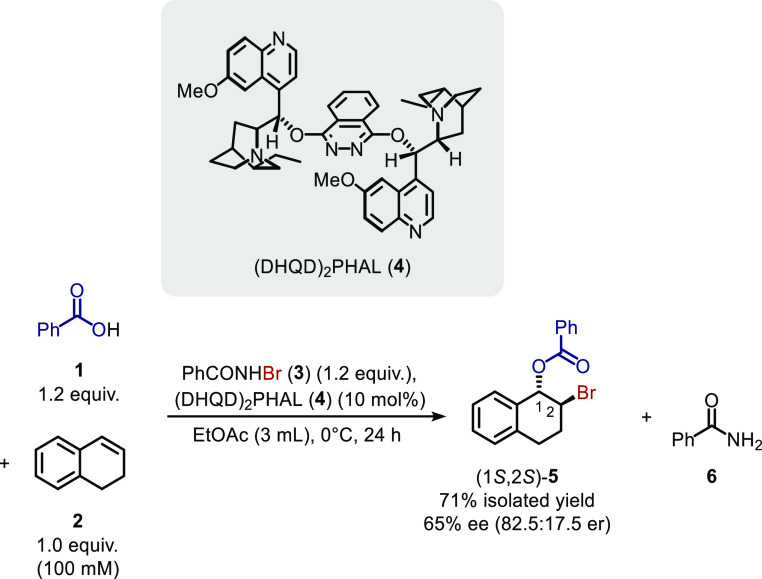
Bromoesterification of Dialin (**2**) to Form 1,2-Bromoester **5** as Reported by Shi *et al*(6a)

**Scheme 2:**
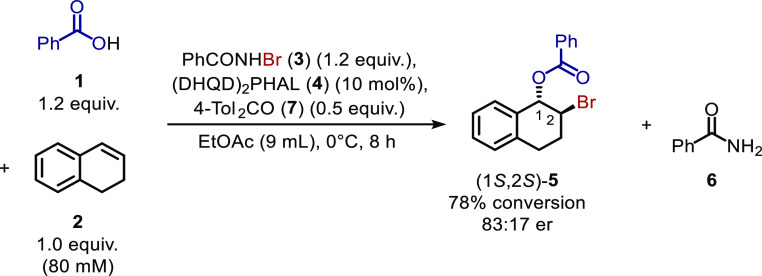
Bromoesterification of Dialin (**2**) to Form 1,2-Bromoester **5** Using Modified Conditions

Dialin (**2**) bromobenzoylation with PhCO_2_H (**1**) and PhCONHBr (**3**) catalyzed by (DHQD)_2_PHAL (**4**), as reported by Shi *et al.*,^6a^ was selected as a representative asymmetric intermolecular alkene bromoesterification reaction (Scheme 1). In Shi *et al.*’s report, bromoester **5** was obtained when 1.2 equiv of PhCONHBr and benzoic acid were added successively to a mixture of dialin (100 mM) and (DHQD)_2_PHAL (10 mol %) in EtOAc at 0 °C with stirring for 24 h. Some slight modifications were made to this procedure to predispose the reaction toward a kinetic analysis: (i) solid reactants and the catalyst were added as stock solutions, (ii) the reaction concentration was reduced (while maintaining the previously defined stoichiometry) to aid with the solubility of the PhCONHBr, which had been observed to precipitate from a stock solution on cooling, (iii) the addition sequence was changed such that reactants and catalyst were added to the PhCONHBr, which assisted with experimental reproducibility, and (iv) 4,4′-dimethylbenzophenone (**7**) was added into each reaction as an internal standard. HPLC analysis of quenched aliquots withdrawn periodically from the reaction was used as the main approach for monitoring the formation of bromoester **5**. Moreover, ^1^H NMR spectroscopy could be used in addition to HPLC as an orthogonal reaction monitoring approach and thereby validated the HPLC method. Using the specified conditions, the reaction was performed with *ex situ* monitoring (Scheme 2). Although Shi *et al.* quote a reaction time of 24 h, the results from this experiment (Figure 1) immediately demonstrated that over half of the reaction occurs in less than 1 h, with the conversion to bromoester **5** plateauing at 78% after 8 h. Pleasingly, we also obtained the same level of enantioselectivity as Shi *et al.* which did not vary over time.

**Figure 2:**
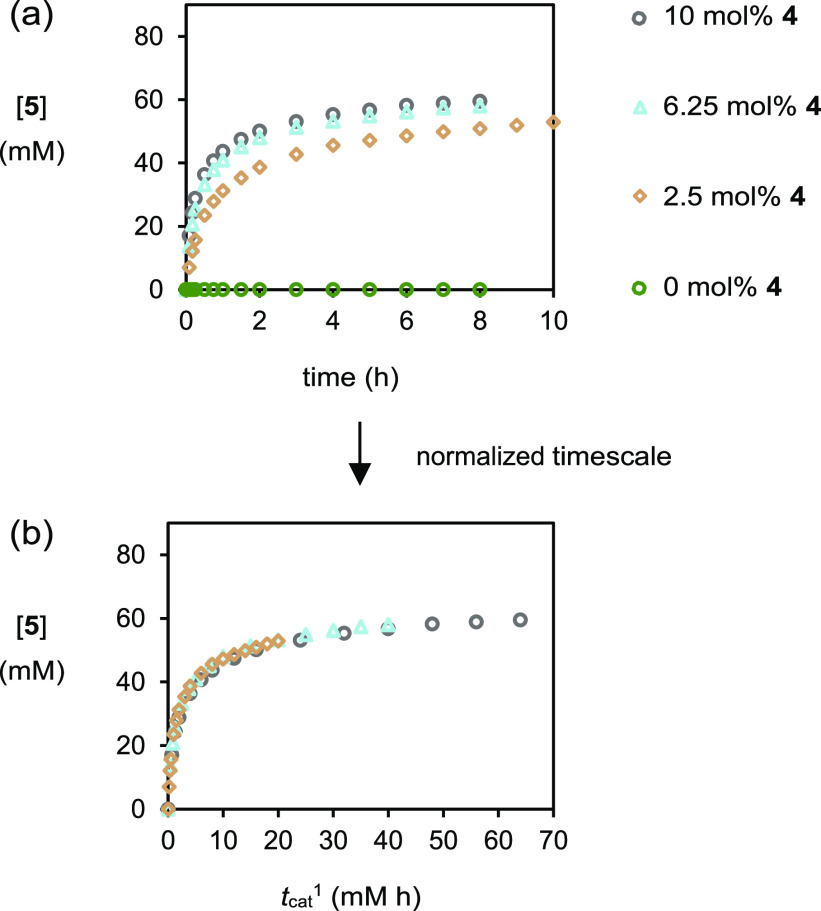
Plot of [**5**] *vs* time in experiments of varying catalyst loading as monitored by HPLC methods. a. (i) ○ (gray), as for Figure 1. (ii) △ (blue), as for Figure 1 with [(DHQD)_2_PHAL]_0_ = 5 mM. (iii) ◇ (brown), as for Figure 1 with [(DHQD)_2_PHAL]_0_ = 2 mM. (iv) ○ (green), as for Figure 1 with [(DHQD)_2_PHAL]_0_ = 0 mM. b. a transformed using a normalized timescale set to first-order: *t*_cat_^1^ = Σ[cat]_0_^1^×(*t*_1_ – *t*_*i*–1_).

Given that the reaction was initially relatively fast, it was decided to see the effect of reducing the catalyst loading. When the catalyst loading was dropped from 10 mol % (8 mM) to 6.25 mol % (5 mM), there was a very small change in the reaction profile (Figure 2a). Further reduction of the catalyst loading to 2.5 mol % (2 mM) reduced the rate of reaction (Figure 2a) but still returned bromoester **5** in an appreciable 66% conversion after 10 h. In addition, a control experiment without a catalyst established the absence of any significant uncatalyzed background reaction to form bromoester **5**. Using variable time normalization analysis,^11^ a first-order dependence in catalyst was determined as shown by the overlaying profiles where the normalized timescale is raised to a power of one (Figure 2b); this is in agreement with an asymmetric alkene chlorolactonization model in the literature.^12^ Across all these experiments, the er again did not change during the reactions, but a slight decrease of 83:17 to 82:18 to 80:20 was observed on reducing the catalyst loading from 10 to 6.25 to 2.5 mol %; this decrease is unexpected, given that no bromoester **5** was observed in the experiment with no catalyst, and requires discussion (*vide infra*). Different excess experiments^11,13^ elucidated that the reaction is first-order in PhCONHBr (**3**) and dialin (**2**),^a^ and zeroth order in the internal standard 4,4′-dimethylbenzophenone (**7**). The order in benzoic acid (**1**), of temporal concentration originally defined as [**1**] = [**1**]_0_ – 2[**4**] – [**5**] (the 1:2 salt of the catalyst with benzoic acid has previously been crystallized^12^ and most likely represents the catalyst resting state, hence two times the concentration of **4** was subtracted), could not be determined at this stage due to a lack of curve overlay (*vide infra*); results are given in the Supporting Information.

**Figure 3:**
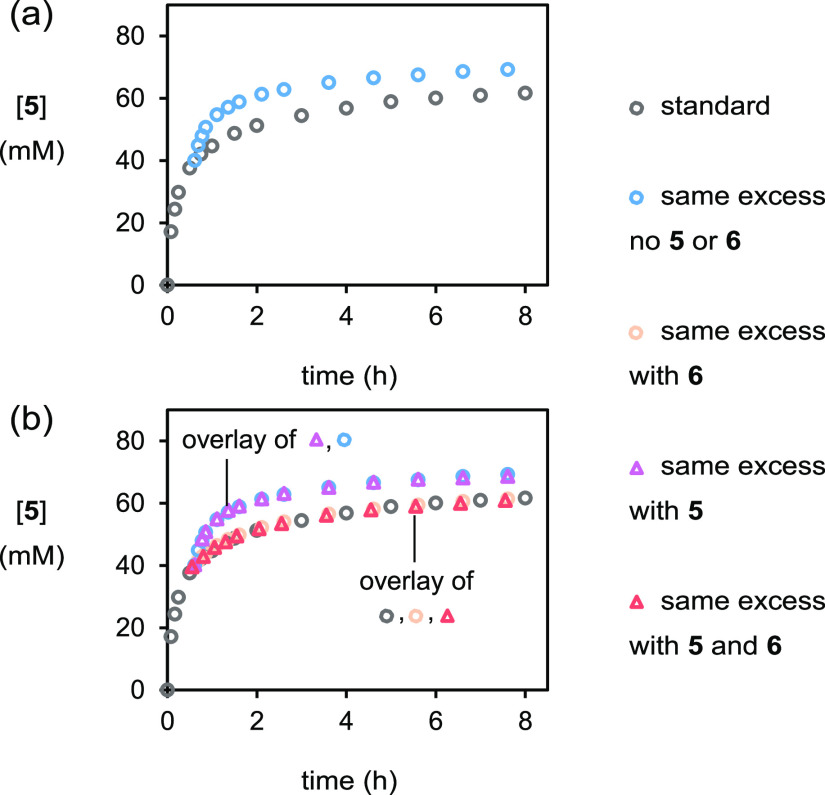
Plot of [**5**] *vs* time in same excess experiments as monitored by HPLC methods. Some datapoints may not be visible due to the curve overlay. a. (i) ○ (gray), as for Figure 1. (ii) ○ (blue), [dialin]_0_ = 40 mM, [PhCO_2_H]_0_ = 56 mM, [PhCONHBr]_0_ = 56 mM and [(DHQD)_2_PHAL]_0_ = 8 mM. b. (i) ○ (gray), as for Figure 1. (ii) ○ (blue), as for a. (iii) ○ (orange), [dialin]_0_ = 40 mM, [PhCO_2_H]_0_ = 56 mM, [PhCONHBr]_0_ = 56 mM, [(DHQD)_2_PHAL]_0_ = 8 mM, and [benzamide]_0_ = 40 mM. (iv) △ (pink), [dialin]_0_ = 40 mM, [PhCO_2_H]_0_ = 56 mM, [PhCONHBr]_0_ = 56 mM, [(DHQD)_2_PHAL]_0_ = 8 mM, and [**5**]_0_ (83:17 er) = 40 mM. (v) △ (red), [dialin]_0_ = 40 mM, [PhCO_2_H]_0_ = 56 mM, [PhCONHBr]_0_ = 56 mM, [(DHQD)_2_PHAL]_0_ = 8 mM, [benzamide]_0_ = 40 mM, and [**5**]_0_ (83:17 er) = 40 mM.

Although for these reactions the rate is initially rapid, the formation of bromoester **5** slows substantially as the reactions proceed, with unreacted dialin (**2**) persisting in the reaction mixture after 8 h. As such, it was wondered if catalyst deactivation or product inhibition could be responsible for the slow reaction rate in the later stages of these reactions. Using the time-adjusted analysis protocol set forth by Blackmond *et al.*,^10^ the same excess experiment at the half-way point of the experiment in Figure 1 was performed ([dialin]_0_ = 40 mM, [PhCO_2_H]_0_ = 56 mM, [PhCONHBr]_0_ = 56 mM, and [(DHQD)_2_PHAL]_0_ = 8 mM) (Figure 3a). When the concentration profile of this experiment was time-adjusted and a 40 mM increase to the concentration of the product bromoester **5** was applied (the amount of bromoester that would have been formed at the half-way point in the higher concentration experiment), the two profiles did not overlay, with instead the lower concentration same excess experiment proceeding more rapidly. This indicated that either the catalyst was deactivating during the experiment or the product(s) were inhibiting the reaction. Three further same excess experiments (see Figure 3 caption for starting concentrations) were then performed with the bromoester **5** and or byproduct benzamide (**6**) added to simulate their concentrations at the half-way point of Figure 1: with amide **6** added, with bromoester **5** added, and with both bromoester **5** and amide **6** added (Figure 3b). For the first and third experiments, the time-adjusted lower concentration profile exactly overlaid with the higher concentration profile. For the second experiment, the resulting profile overlaid with the identical experiment without bromoester **5** added. Taken together, these experiments therefore demonstrate that the reaction is inhibited only by amide **6** and not bromoester **5**. Furthermore, the experiments establish that no catalyst deactivation occurred over the reaction course. Until now, byproduct inhibition has not previously been reported in an alkene halofunctionalization reaction, and this may explain why particular reactions of this class suffer from low catalytic activity.^1c^ For all same excess experiments, the er did not change with time and was the same as shown in Figure 1.

**Figure 4:**
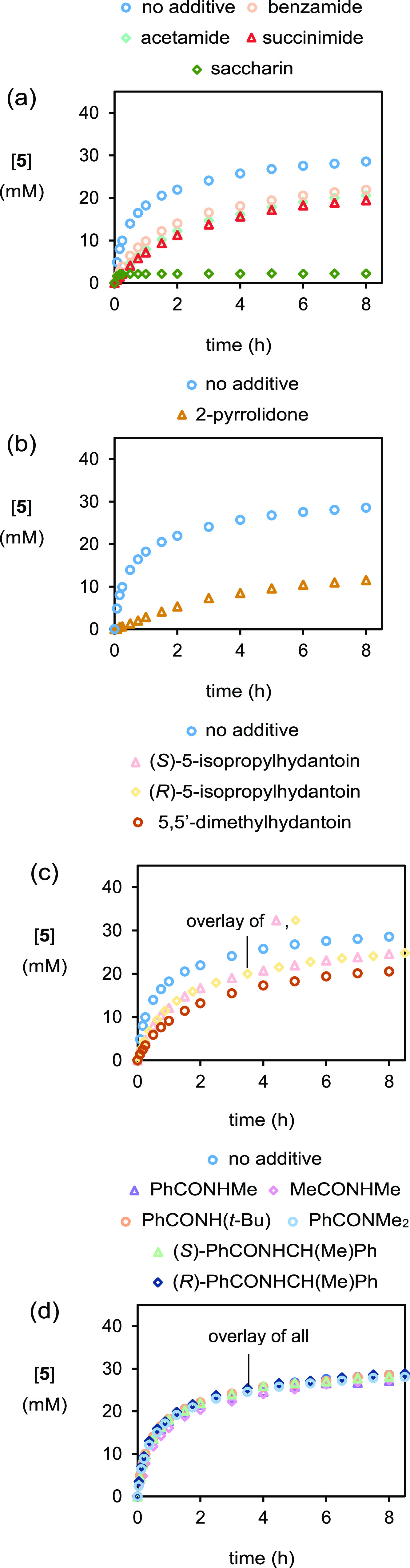
Plot of [**5**] *vs* time in experiments with different byproducts added as monitored by HPLC methods. Some datapoints may not be visible due to curve overlay. (i) ○ (blue), as for Figure 3a. a. (ii) ○ (orange), as for Figure 3b. (iii) ◇ (cyan), with added [acetamide]_0_ = 40 mM. (iv) △ (red), with added [succinimide]_0_ = 40 mM. (v) ◇ (green), with added [saccharin]_0_ = 40 mM (heterogeneous). b. (ii) △ (orange), with added [2-pyrrolidone]_0_ = 40 mM. c. (ii) △ (pink), with added [(*S*)-5-isopropylhydantoin]_0_ = 40 mM. (iii) ◇ (yellow), with added [(*R*)-5-isopropylhydantoin]_0_ = 40 mM. (iv) ○ (brown), with added [5,5′-dimethylhydantoin]_0_ = 40 mM. d. (ii) △ (purple), with added [PhCONHMe]_0_ = 40 mM. (iii) ◇ (pink), with added [MeCONHMe]_0_ = 40 mM. (iv) ○ (orange), with added [PhCONH(*t*-Bu)]_0_ = 40 mM. (v) ○ (blue), with added [PhCONMe_2_]_0_ = 40 mM. (vi) △ (green), with added [(*S*)-PhCONHCH(Me)Ph]_0_ = 40 mM. (vii) ◇ (blue), with added [(*R*)-PhCONHCH(Me)Ph]_0_ = 40 mM.

**Figure 5:**
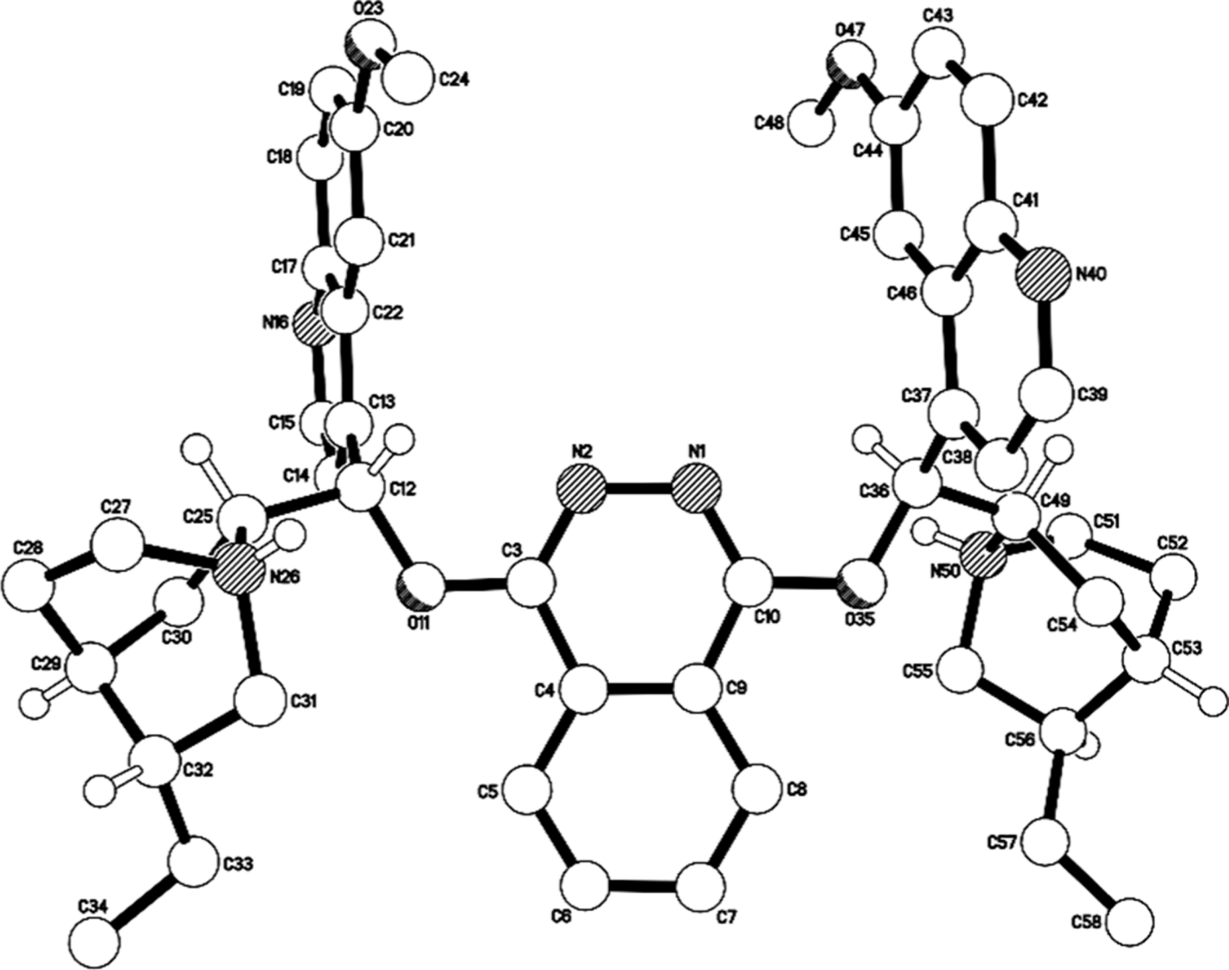
X-ray molecular structure of the salt **4**•(saccharin)_2_ (**8**) with saccharin anions omitted.

**Figure 6:**
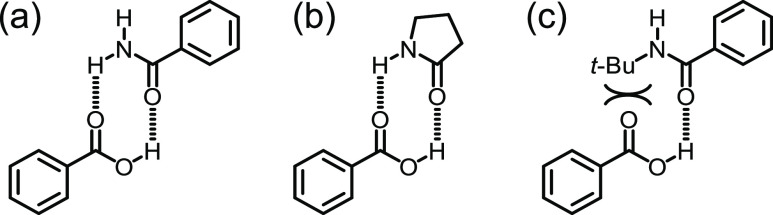
Proposed heterodimerization with double hydrogen bonding of benzoic acid with (a) 1° amide benzamide and (b) 2° cyclic amide 2-pyrrolidone. Heterodimerization with double hydrogen bonding is unavailable with (c) 2° acyclic amide PhCONH(*t*-Bu).

**Scheme 3:**
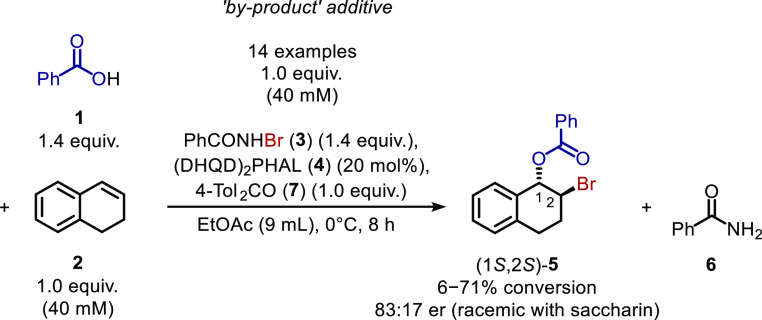
Bromoesterification of Dialin (**2**) to Form 1,2-Bromoester **5** with Added Byproducts

With this system established, it was realized that it could be exploited to examine the possible inhibitory effect of byproducts from different bromenium sources without the need to establish new conditions. Accordingly, experiments in which different putative bromenium ion byproducts were added at a 40 mM concentration were performed (Scheme 3). If these reactions are slower than the reaction without an added byproduct, then the byproduct is contributing toward the inhibition. On the other hand, an overlay of the profiles with and without the byproduct would indicate that no inhibition brought about by the byproduct is taking place. First, experiments with acetamide and succinimide added were performed, which are byproducts from the common bromenium ion sources *N*-bromoacetamide and *N*-bromosuccinimide (Figure 4a). When these byproducts were added, a slower rate of reaction resulted with a similar extent of inhibition to that of benzamide. Saccharin (aq. p*K*_a_ 1.6)^14^ addition resulted in the immediate precipitation of a 1:2 salt of the catalyst and saccharin (**8**), as confirmed by X-ray diffraction measurements following recrystallization from MeOH/hexane (Figure 5). Since the catalyst had been removed from this system by salt formation and precipitation, only a small amount of (essentially racemic) bromoester **5** formed at the onset of the reaction, but no further product was formed over time (Figure 4a). *Cyclic* secondary amide 2-pyrrolidone resulted in the very slow formation of bromoester **5**, indicating strong inhibition by this species (Figure 4b). Addition of 5,5′-dimethylhydantoin, the byproduct of 1,3-dibromo-5,5-dimethylhydantoin, also slowed the formation of bromoester **5** (Figure 4c). This is in direct contrast to a halogen-bond catalyzed bromocarbocylization in which a rate acceleration was observed as hydantoin was liberated.^15^ (*S*)- and (*R*)-5-Isopropylhydantoins, which have previously been utilized as the dichlorenium analogues in an asymmetric alkene chlorolactonization reaction,^16^ inhibited the reaction moderately with no match-mismatch effect observed (Figure 4c). In this case, the lack of a match-mismatch effect suggests that the inhibition is brought about by the interaction of the byproduct with an achiral moiety. Conversely, *acyclic* secondary amides such as PhCONHMe, MeCONHMe, PhCONH(*t*-Bu), and chiral (*S*)- and (*R*)-PhCONHCH(Me)Ph, or a tertiary amide, PhCONMe_2_, did not contribute to the inhibition since the concentration profiles all overlaid (Figure 4d) with the curve with no additive. These results suggest that byproducts with *syn*-periplanar CO and NH groups (*i.e.*, primary amides and cyclic secondary amides) are potent reaction inhibitors, where it is hypothesized that the byproduct heterodimerizes with benzoic acid, thereby sequestering the nucleophile (Figure 6).^17^ Supporting this proposal, Chaudhari and Suryaprakash^18^ have determined the benzoic acid–benzamide association constant to be 22 M^–1^ in CDCl_3_ at 298 K by ^1^H NMR titration experiments. To the best of our knowledge, the interaction of a bromenium ion source byproduct and a nucleophilic component in an alkene halofunctionalization reaction has not previously been implicated. With this finding in hand and with a working hypothesis, attempts were then made to overcome the product inhibition.

**Figure 7:**
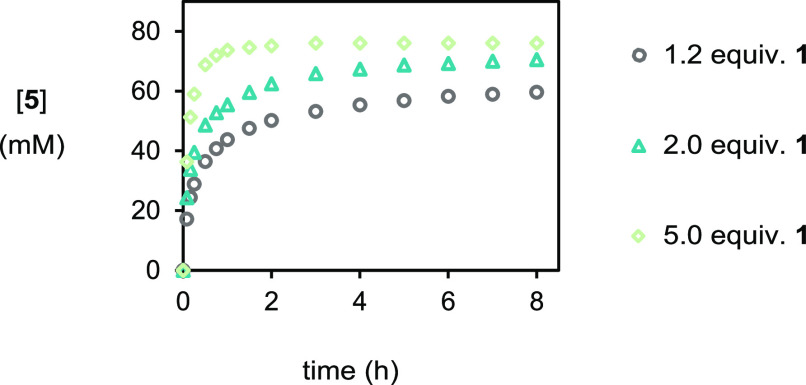
Plot of [**5**] *vs* time in experiments of varying acid concentrations as monitored by HPLC methods. (i) ○ (gray), as for Figure 1. (ii) △ (blue), with [PhCO_2_H]_0_ = 160 mM. (iii) ◇ (green), with [PhCO_2_H]_0_ = 400 mM.

**Figure 8:**
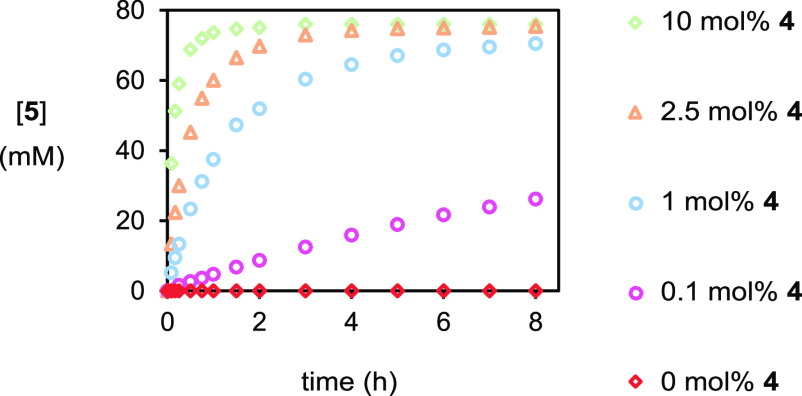
Plot of [**5**] *vs* time in experiments of varying catalyst loading at elevated acid concentrations as monitored by HPLC methods. (i) ◇ (green), as for Figure 7. (ii) △ (orange), with [(DHQD)_2_PHAL]_0_ = 2 mM. (iii) ○ (blue), with [(DHQD)_2_PHAL]_0_ = 0.8 mM. (iv) ○ (pink), with [(DHQD)_2_PHAL]_0_ = 0.08 mM. (v) ◇ (red), with [(DHQD)_2_PHAL]_0_ = 0 mM.

**Scheme 4:**
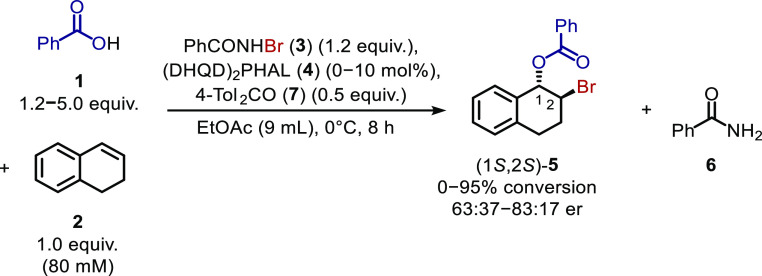
Bromoesterification of Dialin (**2**) to Form 1,2-Bromoester **5** with Varying Benzoic Acid (1.2–5.0 equiv) and Catalyst Loading (0.1–10 mol %)

Two solutions were envisaged to resolve the reaction inhibition by the benzamide byproduct. First, if the number of equiv of benzoic acid is increased, then the effect of the acid-amide heterodimerization should be mitigated. Accordingly, in a series of experiments (Scheme 4), when benzoic acid concentration was increased from 96 (1.2 equiv) to 160 (2.0 equiv) to 400 mM (5.0 equiv), bromoester **5** formed more rapidly and reached higher limiting concentrations after 8 h (Figure 7). Gratifyingly, in the 400 mM benzoic acid experiment, dialin was fully consumed, which had not been observed in any prior experiment. Using the enhanced reaction rate at a benzoic acid concentration of 400 mM, the effect of lowering the catalyst loading from 10 to 2.5 to 1 mol % was reinvestigated (Figure 8). Although lowering catalyst loading reduced the initial reaction rate, conversion to bromoester **5** remained largely unchanged after 8 h with (minimal) depletion of the er from 80:20 to 78:22 to 76:24. On lowering the catalyst loading further to 0.1 mol %, however, the reaction slowed down significantly, and bromoester **5** was returned in a low er of 63:37; thus, 1 mol % effectively marks the limit of catalyst loading in this system to produce an acceptable er. To the best of our knowledge, this is the first intermolecular catalytic asymmetric bromofunctionalization reaction to function efficiently at 1 mol % loading,^19^ albeit with a slightly reduced er compared with Shi *et al.* Since there is no background formation of bromoester **5** in the absence of catalyst (Figure 8), and the addition of benzamide (**6**) to a reaction without catalyst^b^ did not result in bromoester **5** formation either, it remains difficult to rationalize this drop in er.

Supposing that benzamide sequesters benzoic acid as a 1:1 heterodimer, a first-order dependence of benzoic acid could be determined by overlay of the time-normalized Figure 7 curves when a simple modification to the equation for [**1**] was applied. That is, by subtraction of a further 0.9[**5**] ([**1**] = [**1**]_0_ – 2[**4**] – 1.9[**5**], [**5**] = [**6**] due to the reaction stoichiometry) to account for benzoic acid sequestered by benzamide, a best fit for a first-order relationship was found (see Supporting Information).

**Figure 9:**
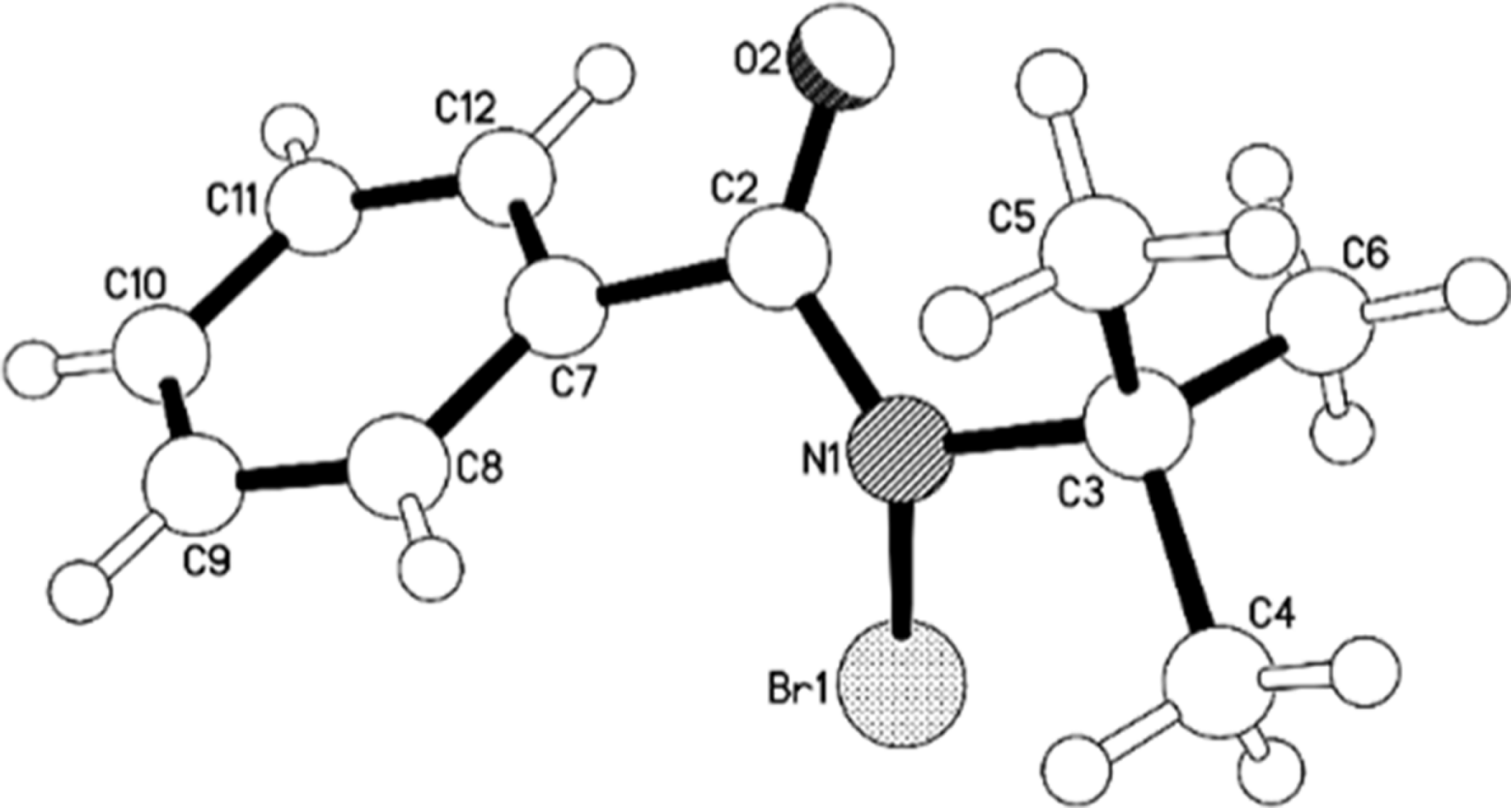
X-ray molecular structure of *N*-alkyl-*N*-bromoamide **9**.

For a second solution to the byproduct inhibition, it was postulated that if a bromenium source is selected with a byproduct that is not inhibitory to the reaction, then the reaction should proceed more quickly, assuming the bromenium source is not significantly slower to react than PhCONHBr. Thus, *N*-alkyl-*N*-bromoamides PhCONBr(*t*-Bu) (**9**)^20^ and PhCONBrMe (**10**), which possess non-inhibitory byproducts as established by prior byproduct addition experiments (Figure 4d), were selected for investigation. *N*-Alkyl-*N*-bromoamides **9** and **10** were synthesized by reaction of the corresponding amides with acetyl hypobromite^20^ freshly prepared from silver acetate and bromine in a solution of benzotrifluoride;^21^^,^^c^ this is a modification from the original preparation in which hepatotoxic and regulatory restricted carbon tetrachloride^22^ was used. *N*-Alkyl-*N*-bromoamide **10** was a viscous, difficult-to-transfer oil, but **9** was a solid, pleasingly forming crystalline pale-yellow plates after evaporation of the benzotrifluoride. X-ray diffraction measurements (Figure 9) determined that *N*-alkyl-*N*-bromoamide **9** possesses a C=O bond length of 1.211(5) Å and a C–N bond length of 1.390(5) Å; these bonds are shortened by 0.013(6) Å and elongated by 0.052(6) Å, respectively, when compared to PhCONH(*t*-Bu) (**11**). Furthermore, *N*-alkyl-*N*-bromoamide **9** possessed substantial nitrogen pyramidalization with a Winkler–Dunitz^23^ χ_N_ value of 40.52° compared to that of 4.99° in the parent amide. This, to the best of the authors’ knowledge, represents the first X-ray structure of an acyclic *N*-brominated secondary amide.

**Scheme 5:**
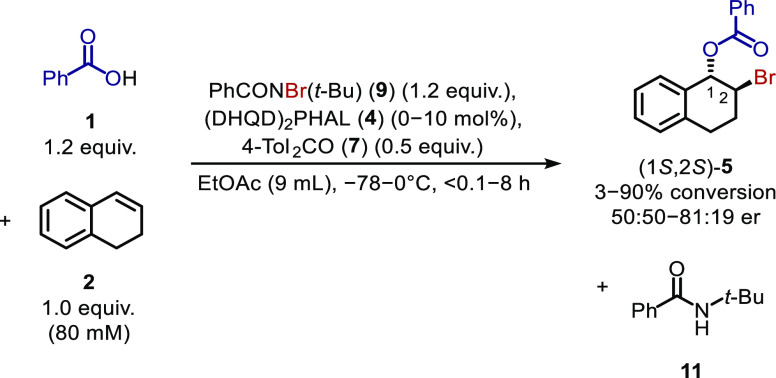
Bromoesterification of Dialin (**2**) to Form 1,2-Bromoester **5** Using *N*-Alkyl-*N*-Bromoamide **9** and 0.1–10 mol % Catalyst Loading

Accordingly, *N*-alkyl-*N*-bromoamide **9** was then employed using the conditions of Scheme 5 at 0 °C and 10 mol % (DHQD)_2_PHAL loading. Much to our delight, this reaction was dramatically fast and effectively complete in 5 min, delivering the bromoester **5** in an overall 90% conversion and 74:26 er (Table 1, entry 1). Further reduction of the catalyst loading to 1 mol % did not significantly impact the reaction time and gave bromoester **5** in 86% conversion with a reduced 63:37 er (Table 1, entry 2). When (DHQD)_2_PHAL loading was only 0.1 mol %, the reaction was complete in under 1 h (Table 1, entry 3), albeit bromoester **5** was returned in 79% conversion and close to racemic (56:44 er). Similarly to reactions with PhCONHBr (**3**), the fall in er with catalyst loading did not appear to result from an uncatalyzed racemic background reaction, as a reaction with no catalyst only gave bromoester **5** in very low conversion after 8 h (Table 1, entry 4). With 1 mol % established as a viable catalyst loading, the effect of lowering the temperature was then investigated with the aim of increasing the enantioselectivity (Table 1). When the 1 mol % catalyst loading reaction was cooled to −15 °C, the reaction was complete after 1 h and in an improved er of 71:29 (Table 1, entry 5), while at −30 °C, an er of 74:26 was obtained after 2 h (Table 1, entry 6). At −78 °C, the er under these conditions was further improved to 81:19—directly comparable to the original level of enantioselectivity reported by Shi *et al.* (Scheme 1), but the reaction was far slower and incomplete after 8 h (Table 1, entry 7).

**Table 1:** Experiments with *N*-Alkyl-*N*-Bromoamide **9** Varying Catalyst Loading^a^

entry	*T* (°C)	catalyst loading (%)	conversion (%)	time (h)	er (5)
1	0	10	90	<0.1	74:26
2	0	1	86	<0.2	63:37
3	0	0.1	79	<1	56:44
4	0	0	3	8	50:50
5	–15	1	84	1	71:29
6	–30	1	80	2	74:26
7	–78	1	18	8	81:19

a Conversion values (100 × [**5**]/[**2**]_0_) and er values are given as monitored by HPLC methods.

**Scheme 6:**
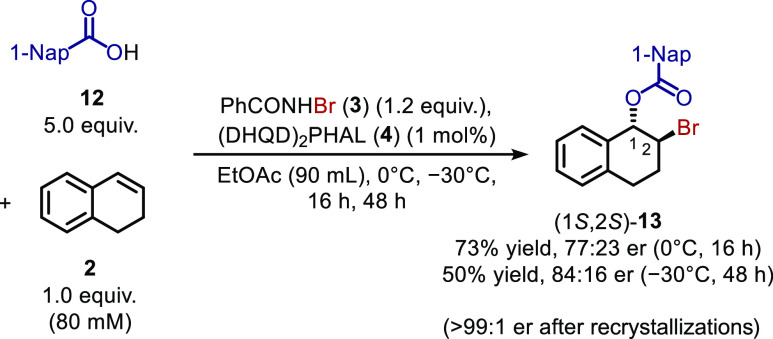
Bromoesterification of Dialin (**2**) to Form 1,2-Bromoester **13** with 1 mol % Catalyst Loading

Since *N*-alkyl-*N*-bromoamide **9** involves the use of an expensive silver reactant in its synthesis, the use of excess carboxylic acid is the more user-friendly approach for overcoming byproduct inhibition. When the catalyst loading is lowered, however, bromoester **5** is obtained in an er lower than Shi *et al.*’s original reaction (Scheme 1). Thus, to enhance the er of the bromoester obtained *post*-reaction and to improve the general utility of asymmetric alkene halofunctionalization reactions, a recrystallization of the bromoester to homochirality was sought.^24^ Since bromoester **5** is an oil, solid bromoester **13** was targeted as a product to be formed using excess 1-naphthoic acid (**12**) rather than benzoic acid. Further, to prevent waste of the acid reactant and catalyst, it was aimed design a procedure for the recycling of these compounds. Following reaction with excess 1-naphthoic acid, aqueous work-up, and column chromatography, bromoester **13** was obtained (Scheme 6)^d^ in 77:23 er.^e^ The (DHQD)_2_PHAL catalyst could be recovered (89%) by flushing the silica column with ethanol. Unreacted, excess 1-naphthoic acid could be quantitatively recycled during the work-up by forming its aqueous soluble lithium salt with an aqueous Li_2_CO_3_ solution and reacidification. Moreover, an iterative recrystallization regime (see Supporting Information) of the bromoester product from methyl *tert*-butyl ether enabled us to retrieve crystals of >99:1 er. This regime involved the collection of the mother liquor of enhanced optical purity and discarding of the crystallized racemate; the recrystallization was then repeated until enantiopure crystals were obtained.^24^ X-ray diffraction measurements of the crystals (see Supporting Information) gave proof of the absolute stereochemistry of (1*S*,2*S*)-bromoester **13** and are in agreement with the assignment by Shi *et al.*(6a) Analogously, bromoester **13** in 84:16 er^e^ was synthesized at −30 °C,^d^ and in two successive recrystallizations, crystals of >99:1 er were obtained (26% recrystallization yield).

## Conclusions

In conclusion, it has been demonstrated that a catalytic intermolecular asymmetric alkene bromoesterification reaction is inhibited by byproducts from stoichiometric bromenium ion sources derived from *primary* and *cyclic* secondary amides. This represents the first time byproduct inhibition has been observed in an alkene halofunctionalization reaction and shows the value of a kinetic profiling approach. Two approaches to surmounting the inhibition are as follows: either increasing equiv of the acid nucleophile, thereby mitigating heterodimerization with the byproduct, or opting to use a bromenium source with a non-inhibitory byproduct (*i.e.*, an *acyclic* secondary amide, which cannot heterodimerize by double hydrogen bonding with the acid). Using both approaches, it was possible to reduce the (DHQD)_2_PHAL loading from 10 to 1 mol % without lengthening the reaction time. The er of bromoester products fell as (DHQD)_2_PHAL loading was lowered but could be recovered at a lower temperature once inhibition had been circumvented. A recrystallization regime of *post*-reaction material from methyl *tert*-butyl ether enabled a bromoester to be obtained in >99:1 er. It is expected that byproduct inhibition will be operative in other alkene haloesterification reactions using standard halenium ion sources and that these findings to surmount it will translate to other systems.^25^

## Experimental Section

### General Experimental Methods

All reagents and commercial grade solvents were used without further purification unless otherwise specified. All reactions were performed in oven-dried glassware under a positive pressure of nitrogen, unless otherwise stated. Column chromatography and filtrations in the sampling procedure in kinetic runs were performed using Geduran Si 60, particle size 40–63 μm. Analytical thin layer chromatography (TLC) was performed on Merck Kieselgel 60 F_254_ pre-coated aluminum-backed plates and was visualized by irradiation with UV light (254 or 366 nm) or staining with potassium permanganate solution.

^1^H NMR and ^13^C{^1^H} NMR spectra were recorded on a Bruker AV-400. ^1^H NMR spectra were recorded at 400 MHz. ^13^C{^1^H} NMR spectra were recorded at 101 MHz. All chemical shifts (δ) are expressed in ppm (parts per million) relative to the residual solvent peak (deuterated chloroform) unless stated otherwise. Abbreviations for multiplicities are: s, singlet; d, doublet; t, triplet; q, quartet; m, multiplet. Fourier transform infrared (IR) spectra were recorded neatly using an ATR-IR spectrometer. Mass spectra were recorded by the Imperial College Department of Chemistry Mass Spectroscopy Service. Melting points were recorded on a Stuart Melting Point Apparatus (SMP20) or an OptiMelt (MPA100). Optical rotations were measured on an ADP 440+ polarimeter with a path length of 0.5 dm, using the d-line of sodium; concentrations (*c*) are quoted in g/100 mL. X-ray crystallography studies were conducted by the Imperial College Department of Chemistry X-ray Crystallography Facility using an Agilent Xcalibur PX Ultra A diffractometer. All kinetic bromoesterification experiments were repeated at least once, and the resulting profiles were averaged between runs.

### Substrates and Reagents

Dialin (**2**) was synthesized following the procedure of Kumar^26^ and was freeze–pump–thaw degassed and stored under nitrogen in a Young ampoule in the dark; this reduced the tendency of the liquid to turn yellow. (DHQD)_2_PHAL (**4**) was synthesized following the procedure of Sharpless^27^ using synthesized or commercially purchased 1,4-dichlorophthalazine and dihydroquinidine hydrochloride. On one occasion, the synthesized (DHQD)_2_PHAL was contaminated with 4-chlorophthalazin-1(2*H*)-one (**14**), which is a common impurity in 1,4-dichlorophthalazine according to Hirsch and Orphanos,^28^ and could be purified by flash column chromatography (100% EtOAc, then 50% EtOAc and EtOH, with 0.2 mL NEt_3_ per 10 mL). Attempted recrystallization of the crude (DHQD)_2_PHAL from hot EtOAc/hexane provided instead 4-chlorophthalazin-1(2*H*)-one, which could be analyzed by single-crystal X-ray diffraction. (*R*/*S*)-5-Isopropylhydantoin (both 98:2 er) were synthesized following the procedure of Suzuki^29^ and enantiopurity was assessed using polarimetry: [α]_D_^26^ = +72.7 (*c* = 0.96, EtOH) and [α]_D_^26^ = −89.7 (*c* = 0.96, EtOH), respectively, and chiral SFC. (*R*/*S*)-PhCONHCH(Me)Ph were synthesized using a MeSi(OMe)_3_-mediated direct amidation protocol^30^ and recrystallized from *n*-hexane/chloroform; enantiopurity (>99:1 er) was assessed using HPLC with a CHIRALPAK-AD stationary phase. PhCONH(*t*-Bu) (**11**) was prepared following the procedure of Denmark^31^ and recrystallized from hot ethanol.

### Preparation of *N*-Bromoamides

#### *N*-Bromobenzamide (**3**)

Using a modified procedure of Shizuo,^32^ NaBr (6.90 g, 67.1 mmol) was added slowly to a solution of benzamide (**6**) (12.1 g, 100 mmol) and sodium bromate (7.60 g, 50.4 mmol) in AcOH (49 mL), H_2_O (21 mL), and concentrated H_2_SO_4_ (4 mL) under stirring at RT. After 20 min, the reaction was stopped stirring and H_2_O (40 mL) was added to induce precipitation of PhCONHBr (**3**). Vacuum filtration followed with washing of the solid with H_2_O (40 mL) gave an off-white solid (15.3 g). For removal of trace EtOAc insoluble impurities, the solid was dissolved in EtOAc and dried over MgSO_4_. Filtration followed by slow cooling of the solution to −20 °C gave large colorless crystals (4.73 g, 24%), which were stored in a foil-wrapped vial in a fridge (4 °C). If a greater yield is desired or if the recrystallization fails, then the EtOAc solution can be left to slowly evaporate overnight, providing large colorless crystals (15.3 g, 76%). Alternatively, the EtOAc solution can be concentrated *in vacuo* at RT to retrieve a white powder. Powdered PhCONHBr (**3**) was easier to manipulate and appeared to turn yellow more slowly. PhCONHBr (**3**) slowly turns from white to yellow on storage in a fridge (4 °C) and was reprepared or recrystallized from EtOAc every few weeks to remove this coloration. mp. 124.3 °C (dec.) (lit. 124–126 °C); IR (film) 3065, 1719 cm^–1^;^32^^1^H NMR (400 MHz, acetone-*d*_6_): δ 8.64 (br s, 1H), 7.90–7.87 (m, 2H), 7.56 (tt, *J* = 7.4, 1.3 Hz, 1H), 7.50–7.45 (m, 2H); ^13^C{^1^H} NMR (101 MHz, acetone-*d*_6_): δ 167.7, 133.2, 132.6, 129.4, 128.7. HRMS (APCI^+^) calcd. for C_7_H_7_^79^BrNO (M + H)^+^, 199.9706; found, 199.9702.

#### *N*-Bromo-*N*-(*tert*-butyl)benzamide (**9**)

Using a modified procedure of Schmidt *et al.*,^20^ PhCF_3_ (75 mL) was added to a flask wrapped in foil and AgOAc (2.50 g, 15.0 mmol) was added with stirring (important!). The resultant suspension was then cooled in ice, and Br_2_ (0.76 mL, 15 mmol) was added dropwise. The reaction mixture was stirred in the dark for an additional 15 min at 0 °C, followed by quickly filtering in air over a Celite pad into a dried, one-necked flask wrapped in foil and cooled to 0 °C; afterward, this flask was placed under N_2_. The dark orange acetyl hypobromite solution was then used immediately. PhCONH(*t*-Bu) (**11**) (1.06 g, 6.00 mmol) was added to a flask wrapped in foil, and acetyl hypobromite solution (45 mL, ≤0.2 M in PhCF_3_, ≤9 mmol) was added at RT. After 2 h, the solution was concentrated *in vacuo* at RT, giving pale yellow plates (1.37 g, 89%), which proved suitable for single-crystal X-ray diffraction. mp. 121.8 °C (dec.); IR (film) 1650 cm^–1^; ^1^H NMR (400 MHz, CDCl_3_): δ 7.65–7.63 (m, 2H), 7.45–7.36 (m, 3H), 1.55 (s, 9H); ^13^C{^1^H} NMR (101 MHz, CDCl_3_): δ 177.3, 137.2, 131.0, 128.6, 128.0, 63.6, 28.8; HRMS (APCI^+^) calcd. for C_11_H_16_NO (M + 2H – Br)^+^, 178.1232; found, 178.1227.

#### *N*-Bromo-*N*-methylbenzamide (**10**)

Using the procedure specified above, *N*-bromo-*N*-methylbenzamide was obtained as an orange oil (1.28 g, 100%). IR (film) 1641 cm^–1^; ^1^H NMR (400 MHz, CDCl_3_): δ 7.49–7.37 (m, 5H), 3.48 (s, 3H); ^13^C{^1^H} NMR (101 MHz, CDCl_3_): δ 172.1, 133.1, 130.8, 128.5, 127.8, 46.1.

#### Procedure for Kinetic Run in Figure 1

With stirring in ice, (DHQD)_2_PHAL (**4**) (1.50 mL, 0.048 M EtOAc stock) was added to a vial, followed by PhCO_2_H (**1**) (1.50 mL, 0.576 M EtOAc stock) and 4-Tol_2_CO (**7**) (0.600 mL, 0.600 M EtOAc stock). Dialin (**2**) (94.0 μL) was added to this solution, and a sample (25 μL), *t* (min) = 0, was taken. Separately, PhCONHBr (**3**) (171.6 mg, corrected for aliquot by 3.575/3.600) was added to a two-necked flask with a thermometer. EtOAc (5.4 mL) was added, and the solution was stirred at 500 rpm at RT until complete homogeneity was observed. The PhCONHBr (**3**) solution was cooled in ice and immediately on temperature stabilization, the other solution was transferred rapidly over using a fridge-chilled syringe (12 mL). For details of other kinetic experiments, see the Supporting Information. At each specified time, an aliquot (25 μL) was removed from the reaction and added to a mixture of saturated aqueous Na_2_S_2_O_3_ (200 μL) and MeCN (200 μL). This was shaken horizontally for 30 s, followed by a pressurized filtration of the top layer over Geduran Si 60 (∼120 mg) in a cotton-packed Pasteur pipette into a HPLC vial. MeCN (300 μL) was passed through the silica under pressure, and then the sample was analyzed by achiral reverse-phase HPLC. After analysis, the solvent was removed under air and the resulting residue was redissolved in 95% *n*-hexane, 5% isopropanol (1 mL) then submitted to normal-phase HPLC with a chiral stationary phase. [**5**], [**2**] are determined by HPLC using a SUPELCOSIL LC-18 column; 50% H_2_O, 50% MeCN to 40% H_2_O, 60% MeCN, 1.0 mL/min, 230 nm, 220 nm, *R*_t_ (**2**) = 5.7 min, *R*_t_ (**7**) = 6.8 min, *R*_t_ (**5**) = 11.1 min; er (**5**) was determined by HPLC using a 10 μm CHIRALPAK-AD column; 95% *n*-hexane 5% isopropanol, 1.0 mL/min, RT, 230 nm, 5 μL injection, *R*_t_ [(+)-(1*S*,2*S*)-**5**] = 5.5 min, *R*_t_ [(−)-(1*R*,2*R*)-**5**] = 6.9 min.

#### (±)-(1S*,2S*)-, (+)-(1*S*,2*S*)-, and (−)-(1*R*,2*R*)-2-Bromo-1,2,3,4-tetrahydronaphthalen-1-yl Benzoate [(±)-5, (+)-5, and (−)-5, Respectively]

For HPLC calibration and to obtain an authentic sample of material, racemic bromoester **5** as a colorless oil was prepared using quinuclidine (20 mol %) and NBS at RT in conditions otherwise analogous to Shi *et al.*(6a) IR (film) 1716 cm^–1^; ^1^H NMR (400 MHz, CDCl_3_): δ 8.05–8.03 (m, 2H), 7.57 (tt, 1H, *J* = 7.4, 1.3 Hz), 7.43 (t, 2H, *J* = 7.4 Hz), 7.33–7.27 (m, 2H), 7.21 (t, 2H, *J* = 7.4 Hz), 6.41 (d, 1H, *J* = 4.7 Hz), 4.63 (ddd, 1H, *J* = 7.5, 4.7, 2.9 Hz), 3.14 (ddd, 1H, *J* = 17.3, 8.9, 5.7 Hz), 2.96 (dt, 1H, *J* = 17.3, 5.4 Hz), 2.56 (dddd, 1H, *J* = 14.2, 8.9, 5.7, 2.9 Hz), 2.31 (dq, 1H, 14.2, 5.7 Hz); ^13^C{^1^H} NMR (101 MHz, CDCl_3_): δ 165.9, 136.2, 133.4, 131.7, 130.2, 130.0, 129.0, 128.9, 128.6, 126.8, 73.9, 49.2, 28.1, 26.4. HRMS (APCI^+^) calcd. for C_17_H_15_O_2_ (M – Br)^+^, 251.1067; found, 251.1069; HPLC CHIRALPAK-AD, 99% *n*-hexane, 1% isopropanol, 1.0 mL/min, RT, 230 nm, 5 μL injection, *R*_t_ [(+)-(1*S*,2*S*)-**5**] = 7.1 min, *R*_t_ [(−)-(1*R*,2*R*)-**5**] = 9.7 min. Scalemic (1*S*,2*S*)-**5**, with [α]_D_^25^ = +83.8 (*c* = 0.82, CHCl_3_) (79:21 er), and (1*R*,2*R*)-**5**, with [α]_D_^25^ = −62.2 (*c* = 0.82, CHCl_3_) (76:24 er) (lit.^6a^ [α]_D_ = +96.7 (*c* = 0.82, CHCl_3_) for (1*S*,2*S*)-**5** [87.5:12.5 er]) were prepared using (DHQD)_2_PHAL (**4**) and (DHQ)_2_PHAL, respectively, with NBS at 0 °C in conditions otherwise analogous to Shi *et al*.^6a^

#### (+)-(1*S*,2*S*)-2-Bromo-1,2,3,4-tetrahydronaphthalen-1-yl 1-Naphthoate (**13**)

(DHQD)_2_PHAL (**4**) (56 mg, 0.072 mmol) and dialin (**2**) (0.937 g, 7.20 mmol) were dissolved in EtOAc (90 mL), and the resulting solution was cooled in ice or at −30 °C. PhCONHBr (**3**) (1.73 g, 8.64 mmol) and 1-naphthoic acid (**12**) (6.20 g, 36.0 mmol) were added successively, giving a heterogeneous suspension. Upon stirring in ice for 16 h, the reaction mixture was quenched with saturated aqueous Na_2_S_2_O_3_ (90 mL). The layers were separated, and the organic phase was washed with 0.1 M aqueous Li_2_CO_3_ (360 mL). The aqueous phase was extracted with EtOAc (3 × 90 mL), the combined organics were dried over MgSO_4_, filtered, concentrated, and purified by column chromatography (97% petroleum ether, 3% EtOAc) to afford bromoester **13** (2.02 g, 73%, 77:23 er at 0 °C, or 1.36 g, 50%, 84:16 er at −30 °C) as an oil that gradually solidified to a white solid. mp. 112.5–114.5 °C (MTBE) (lit.^6a^ 150–152 °C); [α]_D_^27^ = +133.2 (*c* = 0.95, CHCl_3_) (>99:1 er) (lit.^6a^ [α]_D_ = +125.9 (*c* = 0.95, CHCl_3_) for (1*S*,2*S*)-**13** [91.5:8.5 er]); IR (film) 1699 cm^–1^; ^1^H NMR (101 MHz, CDCl_3_): δ 8.99 (d, 1H, *J* = 8.6 Hz), 8.15 (d, 1H, *J* = 7.3 Hz), 8.03 (d, 1H, *J* = 8.2 Hz), 7.90 (d, 1H, *J* = 8.2 Hz), 7.64 (t, 1H, *J* = 7.9 Hz), 7.56 (t, 1H, *J* = 7.3 Hz), 7.48–7.41 (m, 2H), 7.33–7.21 (m, 3H), 6.54 (d, 1H, *J* = 4.8 Hz), 4.72 (ddd, 1H, *J* = 7.2, 4.8, 2.9 Hz), 3.23–3.12 (m, 1H), 2.98 (dt, 1H, *J* = 17.4, 5.6 Hz), 2.59 (dddd, 1H, *J* = 14.2, 8.6, 5.6, 2.9 Hz), 2.34 (dq, 1H, *J* = 14.2, 5.6 Hz); ^13^C{^1^H} NMR (101 MHz, CDCl_3_): δ 166.6, 136.2, 134.0, 133.9, 131.9, 131.6, 130.6, 130.1, 129.0, 128.9, 128.7, 128.1, 126.8, 126.6, 126.5, 125.8, 124.6, 74.0, 49.4, 28.3, 26.5; HRMS (APCI^+^) calcd. for C_21_H_17_O_2_ (M – Br)^+^, 301.1223; found, 301.1237. For 1-naphthoic acid (**12**) recovery, the basic aqueous phase was acidified by dropwise addition of HCl (37% *w*/*w* aqueous) until effervescence and white solid formation ceased (pH 10 → 1). EtOAc (90 mL) was added, the homogeneous phases were separated, and the aqueous was extracted with EtOAc (2 × 90 mL). The combined organics were dried over MgSO_4_, filtered, and concentrated *in vacuo* to recover the unreacted 1-naphthoic acid (**12**) (5.10 g, 100% recovery of theoretical). For (DHQD)_2_PHAL (**4**) recovery, after elution of the bromoester **13** from the column, EtOAc was used to first wash the column, then 50% EtOH, 50% EtOAc, with NEt_3_ (0.2 mL) per 10 mL was eluted, enabling recovery of (DHQD)_2_PHAL (**4**) (50 mg, 89% recovery, readily observed by its blue fluorescence at 366 nm) as a yellow oil. (1*S*,2*S*)-**13** was recrystallized from MTBE to obtain essentially homochiral crystals; details are in the Supporting Information.

#### (−)-(1*S*,2*S*)-2-Bromo-1,2,3,4-tetrahydronaphthalen-1-ol (**15**)

DIBAL-H (0.40 mL, 0.70 M in *n*-hexane, 0.28 mmol) was added to a stirred suspension of bromoester **5** or **13** (0.125 mmol) in *n*-hexane (2.5 mL) at 0 °C, and the reaction was stirred for 1 h, during which it became homogeneous. The reaction was quenched by the addition of MeOH (5 drops), diluted with CH_2_Cl_2_ (1.25 mL), H_2_O (2.5 mL), and aqueous H_2_SO_4_ (1.25 mL, 1 M), and was allowed to warm to RT. The layers were separated, and the aqueous phase was extracted with CH_2_Cl_2_ (3 × 5 mL). The combined organics were dried over MgSO_4_, filtered, and concentrated *in vacuo* to yield a residue purified by column chromatography (91% petroleum ether, 9% EtOAc), affording the bromohydrin (**15**) (10.2 mg, 36% from **5**, 23.8 mg, 84% from **13**) as a white solid. mp. 109.7–110.5 °C (CHCl_3_) (lit.^6a^ 89–90 °C); [α]_D_^25^ = −24.6 (*c* = 0.76, CHCl_3_) (75:25 er) (lit.^6a^ [α]_D_ = −36.1 (*c* = 0.78, CHCl_3_) for (1*S*,2*S*)-**15** [90.5:9.5 er]); IR (film) 3233 cm^–1^; ^1^H NMR (400 MHz, CDCl_3_): δ 7.54–7.51 (m, 1H), 7.27–7.22 (m, 2H), 7.12–7.10 (m, 1H), 4.91 (dd, 1H, *J* = 7.0, 4.9 Hz), 4.37 (ddd, 1H, *J* = 9.9, 7.0, 3.2 Hz), 3.03–2.89 (m, 2H), 2.56–2.49 (m, 2H), 2.33–2.24 (m, 1H); ^13^C{^1^H} NMR (101 MHz, CDCl_3_): δ 135.6, 135.1, 128.7, 128.4, 128.2, 126.8, 74.2, 56.3, 29.8, 28.2; HRMS (EI^+^) calcd. for C_10_H_11_^79^BrO (M)^+^, 225.9988; found, 225.9980; HPLC CHIRALPAK-AD, 90% *n*-hexane, 10% isopropanol, 1.0 mL/min, RT, 220 nm, 5 μL injection, *R*_t_ [(+)-(1*R*,2*R*)-**15**] = 7.0 min, *R*_t_ [(−)-(1*S*,2*S*)-1**5**] = 7.9 min.

#### Preparation of 4•(Saccharin)_2_ (**8**)

To (DHQD)_2_PHAL (56.1 mg, 0.072 mmol) in EtOAc (1.5 mL) was added saccharin (65.3 mg, 0.356 mmol) in EtOAc (5.4 mL). Vacuum filtration of the immediately formed precipitate and washing with cold EtOAc provided the salt as a white solid (62.0 mg, 75%). Crystals suitable for single-crystal X-ray diffraction were obtained by slow diffusion of hexane into a solution of **4**•(saccharin)_2_ (**8**) in MeOH. mp. 173.7 °C (dec.); IR (film) 1611 cm^–1^; ^1^H NMR (400 MHz, CDCl_3_): δ 8.50 (d, 2H, *J* = 4.4 Hz), 8.38–8.36 (m, 2H), 7.99 (d, 4H, *J* = 8.8 Hz), 7.67–7.63 (m, 4H), 7.54 (s, 2H), 7.47 (t, 2H, *J* = 7.0 Hz), 7.38–7.26 (m, 8H), 3.83 (s, 6H), 3.72 (dt, 4H, *J* = 27.6, 9.5 Hz), 3.48–3.42 (m, 6H), 2.61 (t, 2H, *J* = 11.2 Hz), 2.07 (br s, 2H), 1.94–1.70 (m, 12H), 1.49–1.41 (m, 2H), 0.98 (t, 6H, *J* = 7.2 Hz); ^13^C{^1^H} NMR (101 MHz, CDCl_3_): δ 172.0, 158.8, 155.7, 147.3, 144.6, 144.2, 140.1, 133.0, 132.8, 132.3, 132.01, 131.99, 126.0, 123.2, 122.9, 122.6, 122.5, 120.1, 117.8, 101.1, 72.7, 59.5, 56.3, 49.8, 49.7, 35.3, 25.9, 24.9, 23.9, 19.8, 11.6; HRMS (ESI^+^) calcd. for C_48_H_56_N_6_O_4_ (M + 2H)^2+^, 390.2176; found, 390.2184.

## Data Availability

The data underlying this study are available in the published article and its Supporting Information. NMR, IR, and MS data underlying this study are openly available at the Imperial College research data repository at DOI: 10.14469/hpc/12349. X-ray crystal data are deposited in the CCDC (CCDC 2246905–2246910).

## Supporting Information Available

Storage and titration of *N*-bromoamides; details of kinetic runs; details of HPLC conditions; details of crystallization of (1*S*,2*S*)-**13**, additional kinetic runs; X-ray crystallography data for **8**, **9**, **11**, (±)-**13**, (+)-**13**, and **14**; HPLC calibration curves; copies of ^1^H and ^13^C{^1^H} NMR spectra; and representative HPLC chromatograms (PDF)

## Footnotes

a When HPLC monitoring was used to determine [**2**], the best overlay was obtained when the order in dialin (**2**) was 0.8. If instead [**2**] was defined as [**2**]_0_ – [**5**], the order in dialin was 1.0.

b When 40 mM of benzamide (**6**) was added into the reaction shown in Figure 2 with no (DHQD)_2_PHAL (**4**), there was negligible bromoester **5** formation after 8 h, which showed that amide **6** did not catalyze a racemic background reaction to form bromoester **5**.

c Benzotrifluoride was selected due to its lack of labile C–H bonds.

d Under these conditions, 1-naphthoic acid (**12**) did not fully dissolve.

e er of bromoester **13** was determined by HPLC analysis with a chiral stationary phase of bromohydrin **15** obtained by DIBAL-H reduction of bromoester **13**.
