# Supplementary material for: Surmounting Byproduct Inhibition in an Intermolecular Catalytic Asymmetric Alkene Bromoesterification Reaction as Revealed by Kinetic Profiling
Source: J Org Chem. 2023 Jun 16;88(13):8904–14. doi: 10.1021/acs.joc.3c00672 (PMC10337038; doi:10.1021/acs.joc.3c00672)
Supplement: Supplementary file 1 — jo3c00672_si_001.pdf [file jo3c00672_si_001.pdf]

Electronic Supporting Information

Surmounting By-Product Inhibition in an Intermolecular Catalytic Asymmetric Alkene Bromoesterification Reaction as Revealed by Kinetic Profiling

D. Christopher Braddock,*[a] Ben M. J. Lancaster,[a] Christopher J. Tighe[b] and Andrew J. P. White[a]

[a] Department of Chemistry, Molecular Sciences Research Hub, Imperial College London, White City Campus, 82 Wood Lane, London W12 0BZ, UK

[b] Department of Chemical Engineering, Imperial College London, South Kensington Campus, Imperial College Road, London SW7 2AZ, UK

Email: c.braddock@imperial.ac.uk, c.tighe@imperial.ac.uk

Cover Page and Contents

Cover Page and Contents: S1

1. Storage and Titration of *N*-Bromoamides; S2

2. Details of Kinetic Runs; S3

3. Details of HPLC Conditions; S5

4. Details of Crystallisation of (1*S*,2*S*)-**13**; S6

5. Additional Kinetic Runs; S7

6. X-Ray Crystallography; S11

7. HPLC Calibration Curves; S18

8. Copies of ^{1}H and ^{13}C{^{1}H} NMR Spectra; S19

9. Representative HPLC Chromatograms; S33

10. References. S35

1. Storage and Titration of *N*-Bromoamides

1.1. Storage

N-Alkyl-*N*-Bromoamides were stored in a fridge (4°C) or freezer (–20°C) in foil-wrapped vials. PhCONBrMe (**10**) was more stable than PhCONBr(*t*-Bu) (**9**) and after 4 months at –20°C only minor changes were observed by ^{1}H NMR monitoring. PhCONBr(*t*-Bu) (**9**) was found to decompose on storage in a sealed air-filled vial in the dark at 4°C to the corresponding amide at roughly 0.5−1% per day. ^{1}H NMR monitoring can be used to follow this process by relative integration of the *t*-Bu groups.

PhCONBr(*t*-Bu) (**9**) was not recrystallised from cold acetone-water as described in the original procedure[1] as an attempt to do this had led to significant decomposition to PhCONH(*t*-Bu) (**11**).

Before bromoesterification experiments using PhCONBr(*t*-Bu) (**9**), the material was assayed by ^{1}H NMR spectroscopy and corrections were made to the mass of material added to account for its decomposition; typical purities were >85%. The presence of amide **11** is unlikely to affect the rate of bromoester **5** formation based on the non-inhibitory nature of amide **11** established in Figure 4(d).

1.2. Iodometric Titration

*PhCONHBr (**3**)*

PhCONHBr (250.0 ± 0.1 mg) was dissolved in AcOH (5 mL) with KI (1.4 g, 8.4 mmol) and H_2O (20 mL), in a conical flask. This solution was then titrated against a standardised solution of aqueous $Na_2S_2O_3$ (99.4 ± 0.3 mM), with addition of starch indicator (2 mL) near the end point. This process was repeated three times, and the calculated concentration of liberated I_2 was averaged to determine an active bromine% of 40.4 ± 0.2% (39.9% theoretical).

*PhCONBr(t-Bu) (**9**)*

Using the same procedure as for **3**, but with AcOH (20 mL), PhCONBr(*t*-Bu) (320.0 ± 0.1 mg) gave an active bromine% of 29.0 ± 0.2% (31.2% theoretical).

*PhCONBrMe (**10**)*

Using the same procedure as for **9**, PhCONBrMe (268.0 ± 0.1 mg) gave an active bromine% of 35.6 ± 0.2% (37.3% theoretical).

2. Details of Kinetic Runs

2.1. Bromoesterification Reaction at 80 mM Dialin (2)

With stirring in ice, $(DHQD)_2PHAL$ (**4**) (1.50 mL, 0.048 M EtOAc stock) was added to a vial, followed by $PhCO_2H$ (**1**) (1.50 mL, 0.576 M EtOAc stock) and 4-Tol_2CO (**7**) (0.600 mL, 0.600 M EtOAc stock). Dialin (**2**) (94.0 µL) was added to this solution and a sample (25 µL), *t* (min) = 0, was taken.

Separately, PhCONHBr (**3**) (171.6 mg, corrected for aliquot by 3.575/3.600) was added to an oven-dried, two-necked flask with a thermometer. EtOAc (5.4 mL) was added, and the solution was stirred at 500 rpm at RT until complete homogeneity was observed.

The PhCONHBr (**3**) solution was cooled in ice and immediately on temperature stabilisation, the other solution was transferred rapidly over using a fridge-chilled syringe (12 mL).

Addition of the reactants and catalyst to a separate solution of PhCONHBr (**3**) was found to be critical for good reproducibility between runs. On some occasions, the runs failed due to precipitation of $(DHQD)_2PHAL•(PhCO_2H)_2$ before addition to the PhCONHBr (**3**) solution.

2.2. Different Catalyst Loading Bromoesterification Reactions

These followed the same procedure as Section 2.1 but with differing volumes (x) of $(DHQD)_2PHAL$ (**4**) stock (0.048 M EtOAc); x = 0.9375 mL for 6.25 mol%, x = 0.375 mL for 2.5 mol%, x = 0 mL for 0 mol%. EtOAc (1.50 mL – x mL) was added before dialin (**2**) to bring the total volume of the vial solution to 3.6 mL.

2.3. Same Excess Bromoesterification Reactions at 40 mM Dialin (2)

With stirring in ice, $(DHQD)_2PHAL$ (**4**) stock (1.50 mL, 0.048 M EtOAc) was added to a vial, followed by $PhCO_2H$ (**1**) stock (0.875 mL, 0.576 M EtOAc), 4-Tol_2CO (**7**) stock (0.600 mL, 0.600 M EtOAc) and (a) EtOAc (0.625 mL) or (b) bromoester **5** stock (83:17 er, 0.625 mL, 0.576 M EtOAc). Dialin (**2**) (47.0 µL) was added to this solution and a sample (25 µL), *t* (min) = 0, was taken.

Separately, PhCONHBr (**3**) (100.1 mg, corrected for aliquot by 3.575/3.600) was added to an oven-dried, two-necked flask with a thermometer. Either (a) EtOAc (5.4 mL) or (b) $PhCONH_2$ (**6**) stock (5.4 mL, 0.066 M EtOAc, corrected for aliquot by 3.575/3.600) was added and the solution was stirred at 500 rpm at RT until complete homogeneity was observed.

The PhCONHBr (**3**) solution was cooled in ice and immediately on temperature stabilisation, the other solution was transferred rapidly over using a fridge-chilled syringe (12 mL).

2.4. Additive Addition Bromoesterification Reactions

These followed the same procedure as Section 2.3 but with the addition of 'by-product' additive stock solutions other than $PhCONH_2$ (**6**) (as 0.066 M EtOAc) and no bromoester **5** stock.

2.5. Different $PhCO_2H$ (1) Loading Bromoesterification Reactions

With stirring in ice, $(DHQD)_2PHAL$ (**4**) (1.50 mL, 0.048 M EtOAc stock) was added to a vial, followed by $PhCO_2H$ (**1**) (1.50 mL, 0.960 M EtOAc stock) and 4-Tol_2CO (**7**) (0.600 mL, 0.600 M EtOAc stock). Dialin (**2**) (94.0 µL) was added to this solution and a sample, *t* (min) = 0, was taken.

Separately, PhCONHBr (**3**) (171.6 mg, corrected for aliquot by 3.575/3.600) was added to an oven-dried, two-necked flask with a thermometer. Either (a) EtOAc (5.4 mL) or (b) $PhCO_2H$ (**1**) stock (5.4 mL, 0.397 M EtOAc, corrected for aliquot by 3.575/3.600) was added and the solution was stirred at 500 rpm at RT until complete homogeneity was observed.

The PhCONHBr (**3**) solution was cooled in ice and immediately on temperature stabilisation, the other solution was transferred rapidly over using a fridge-chilled syringe (12 mL).

The 160 mM $PhCO_2H$ (**1**) reaction used route (a) whilst the 400 mM $PhCO_2H$ (**1**) reaction used route (b).

2.6. Different Catalyst Loading at 400 mM $PhCO_2H$ (1) Bromoesterification Reactions

These followed the same procedure as Section 2.5, route (b), but with differing volumes (*x*) of $(DHQD)_2PHAL$ (**4**) stock (0.048 M EtOAc); *x* = 0.375 mL for 2.5 mol%, *x* = 0.150 mL for 1.0 mol%, *x* = 0.015 mL for 0.1 mol% and *x* = 0 mL for 0 mol%. EtOAc (1.50 mL – *x* mL) was added before dialin to bring the total volume of the vial solution to 3.6 mL.

2.7. Different Catalyst Loading PhCONBr(*t*-Bu) (9) Bromoesterification Reactions at 80 mM Dialin

These followed the same procedure as Section 2.2 but with (a) PhCONBr(*t*-Bu) (**9**) (typically 219.8 mg but varied depending on the content of N−Br versus N−H) instead of PhCONHBr (**3**) and (b) differing volumes (*x*) of $(DHQD)_2PHAL$ (**4**) stock (0.048 M EtOAc); *x* = 1.50 mL for 10 mol%, *x* = 0.150 mL for 1 mol%, *x* = 0.015 mL for 0.1 mol% and *x* = 0 mL for 0 mol%. EtOAc (1.50 mL – *x* mL) was added before dialin (**2**) to bring the total volume of the vial solution to 3.6 mL. Experiments were also performed at the specified temperature.

3. Details of HPLC Conditions

HPLC was used to quantify [**5**] by integration of the peak for the bromoester **5** (R_t ~ 11.1 min) relative to an internal standard, 4,4'-dimethylbenzophenone (**7**) (R_t ~ 6.8 min), and was performed using a 5.0 cm × 4.6 mm SUPELCOSIL LC-18 column equipped with a guard cartridge and a 5 μm UltraShield UHPLC pre-column filter. 4,4'-Dimethylbenzophenone (**7**) was added into each HPLC monitored reaction at a concentration of 40 mM and was confirmed to be zero order by a different excess experiment at 20 mM concentration. HPLC using a 25 cm × 4.6 mm CHIRALPAK-AD column equipped with a CHIRALPAK AD-H guard cartridge was used for chiral analytical HPLC. Retention times (R_t) are reported in minutes.

4. Details of Crystallisation of (1*S*,2*S*)-13

Iterative recrystallisations of bromoester **13** are summarised below. Recrystallisation (represented by an arrow) was achieved by dissolution in hot MTBE, typically at a concentration of 0.06–0.08 g/mL, followed by slow cooling to −20°C yielding crystals (after several hours or days) which were isolated by vacuum filtration; meanwhile, the mother liquor was concentrated *in vacuo*. Some **13** was kept for analysis at each stage, hence the recrystallisation yields are underrepresented. Enantiomeric ratios were quantified by DIBAL-H reduction of bromoester **13** samples to the corresponding bromohydrin **15** followed by HPLC analysis with a chiral stationary phase.

Batch 1, obtained by reaction at 0°C

(1*S*,2*S*)-**13** (1.87 g, 77:23 er) → crystals (0.705 g, 38%, 57:43 er, **XRD**) + mother liquor (1.11 g, 59%, 93:7 er)

mother liquor (0.709 g, 93:7 er) → crystals (51.4 mg, 7%, 59:41 er) + mother liquor (0.632 g, 89%, 97:3 er)

mother liquor (0.428 g, 97:3 er) → **crystals (0.193 g, 45%, >99:1 er)** + mother liquor (0.187 g, 44%, 85:15 er)

10% recrystallisation yield.

Batch 2, obtained by reaction at −30°C

(1*S*,2*S*)-**13** (0.796 g, 84:16 er) → crystals (0.238 g, 30%, 61:39 er) + mother liquor (0.558 g, 70%, 95:5 er)

mother liquor (0.374 g, 95:5 er) → **crystals (0.209 g, 56%, >99:1 er, XRD)** + mother liquor (0.144 g, 39%, 86:14 er)

26% recrystallisation yield.

5. Additional Kinetic Results

Further information on different excess experiments not discussed in detail in the manuscript are given below.

5.1. 4,4'-Dimethylbenzophenone

4,4'-Dimethylbenzophenone (**7**), the internal standard, was determined to be zeroth order due to overlay of the profiles with an unnormalised timescale (Figure S1).

Table S1. Different excess experiment in 4-Tol_2CO.

concentration (mM)	standard	different excess
$[dialin]_0$	80	80
$[PhCO_2H]_0$	96	96
$[PhCONHBr]_0$	96	96
$[(DHQD)_2PHAL]_0$	8	8
$[(4\text{-}Tol_2CO)]_0$	40	20

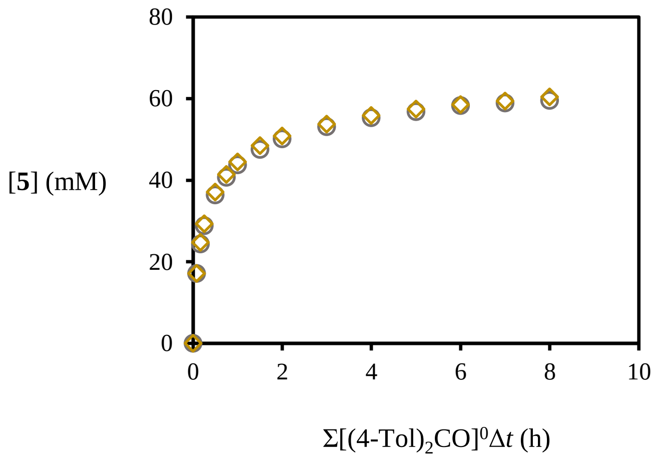

Figure S1. Plot of [**5**] *vs* (un)normalised time (raised to the power of 0) as monitored by HPLC methods: standard (o) and different excess in 4-Tol_2CO (◇) experiments.

5.2. PhCONHBr

PhCONHBr (**3**) was determined to be first order after overlay of the profiles when the normalised timescale was raised to the power of 1.0 (Figure S2). For this, [PhCONHBr] was calculated according to equation 1.

$$[PhCONHBr] = [PhCONHBr]_0 - [\mathbf{5}] \quad (1)$$

Table S2. Different excess experiment in PhCONHBr.

concentration (mM)	standard	different excess
$[dialin]_0$	80	80
$[PhCO_2H]_0$	96	96
$[PhCONHBr]_0$	96	64
$[(DHQD)_2PHAL]_0$	8	8
$[(4\text{-}Tol_2CO)]_0$	40	40

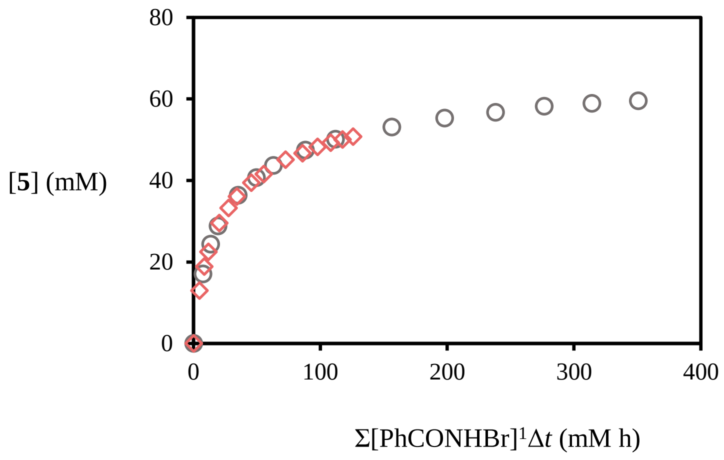

Figure S2. Plot of [**5**] *vs* normalised time raised to the power of 1.0 as monitored by HPLC methods: standard (o) and different excess in PhCONHBr (◇) experiments.

5.3. Dialin

Dialin (**2**) was determined to be first order after overlay of the profiles when the normalised timescale was raised to the power of 1.0 (Figure S3). For this, [dialin] was calculated according to equation 2.

$$[\text{dialin}] = [\text{dialin}]_0 - [\mathbf{5}] \quad (2)$$

Table S3. Different excess experiment in dialin.

concentration (mM)	standard	different excess
$[\text{dialin}]_0$	80	40
$[PhCO_2H]_0$	96	96
$[\text{PhCONHBr}]_0$	96	96
$[(\text{DHQD})_2\text{PHAL}]_0$	8	8
$[(4\text{-Tol}_2\text{CO})]_0$	40	40

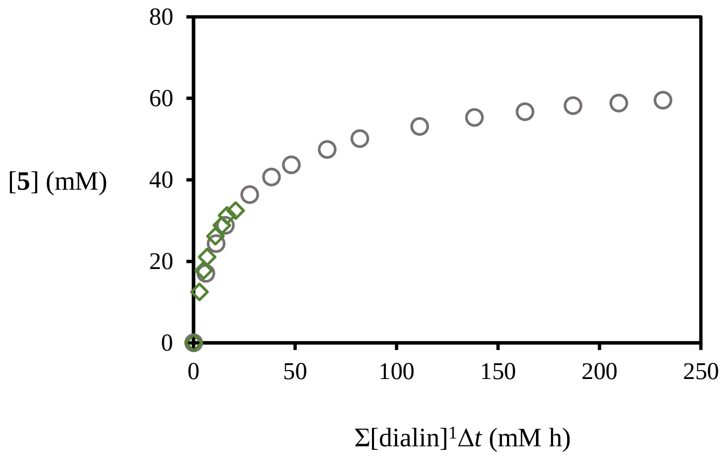

Figure S3. Plot of [**5**] *vs* normalised time raised to the power of 1.0 as monitored by HPLC methods: standard (o) and different excess in dialin (◇) experiments.

5.4. Benzoic acid

Benzoic acid (**1**) was determined to be first order after overlay of the profiles when the normalised timescale was raised to the power of 1.0 (Figure S4). For this, [$PhCO_2H$] was calculated according to equation 3. Since the catalyst resting state is expected to be a salt of benzoic acid (**1**) with $(DHQD)_2PHAL$ (**4**) in a 2:1 ratio, the equation employs a subtraction of two times the concentration of the catalyst.[2] Similarly, we assume that benzamide (**6**) sequesters benzoic acid as a 1:1 heterodimer; the best overlay was found when a correction factor of 0.9 to [**6**] was applied (due to the stoichiometric equation of the reaction, [**6**] = [**5**]).

$$[PhCO_2H] = [PhCO_2H]_0 - [\mathbf{5}] - 0.9[\mathbf{5}] - 2[(DHQD)_2PHAL]_0 \qquad (3)$$

Table S4. Different excess experiments in $PhCO_2H$.

concentration (mM)	standard	different excess	
$[dialin]_0$	80	80	80
$[PhCO_2H]_0$	96	160	400
$[PhCONHBr]_0$	96	96	96
$[(DHQD)_2PHAL]_0$	8	8	8
$[(4\text{-}Tol_2CO)]_0$	40	40	40

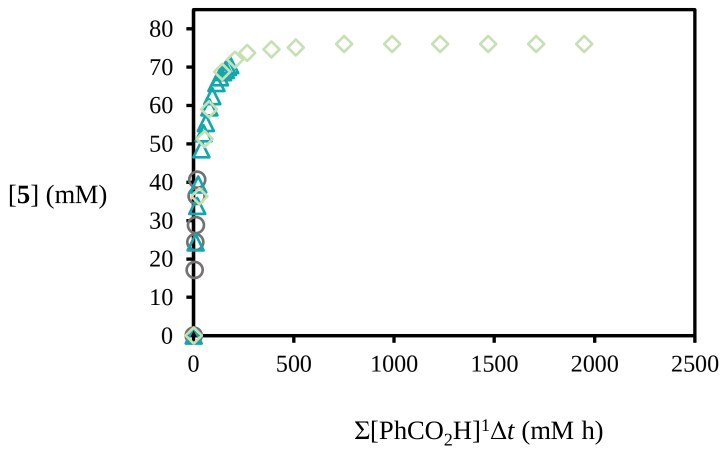

Figure S4. Plot of [**5**] *vs* normalised time raised to the power of 1.0 as monitored by HPLC methods: standard (o) and different excess in $PhCO_2H$ (△ = 160 mM, ◇ = 400 mM) experiments.

6. X-Ray Crystallography

Table S5. Crystal Data, Data Collection and Refinement Parameters for the structures of **8**, **9**, **11**, (±)-**13**, (+)-**13** and **14**.

data	8	9	11	(±)-13
**formula**	$(C_{48}H_{56}N_6O_4)(C_7H_4NO_3S)_2$	$C_{11}H_{14}BrNO$	$C_{11}H_{15}NO$	$C_{21}H_{17}BrO_2$
**solvent**	$2(CH_4O)$	—	—	—
**formula weight**	1209.41	256.14	177.24	381.26
**colour, habit**	colourless needles	pale yellow plates	colourless needles	colourless blocks
**temperature / K**	173	173	173	173
**crystal system**	monoclinic	monoclinic	orthorhombic	triclinic
**space group**	$P2_1$ (no. 4)	$P2_1/c$ (no. 14)	*Pbca* (no. 61)	*P*-1 (no. 2)
a **/ Å**	10.8225(8)	16.5949(6)	8.8334(2)	10.4067(3)
b **/ Å**	28.4373(16)	6.7477(2)	10.1657(2)	11.6025(4)
c **/ Å**	10.9044(6)	10.2051(4)	23.3388(7)	15.2010(5)
**α / deg**	90	90	90	71.693(3)
**β / deg**	110.772(7)	101.319(4)	90	78.254(3)
**γ / deg**	90	90	90	72.294(3)
V **/ Å³**	3137.8(4)	1120.50(7)	2095.76(10)	1648.09(10)
Z	2	4	8	4 [c]
D_c **/ g cm⁻³**	1.280	1.518	1.123	1.537
**radiation used**	Cu-Kα	Cu-Kα	Cu-Kα	Cu-Kα
**μ / mm⁻¹**	1.324	4.739	0.563	3.472
**no. of unique reflns**				
**measured (**R_{int}**)**	11971 (0.0816)	2165 (0.0429)	2021 (0.0298)	6543 (0.0342)
**obs,** $\|F_o\| > 4\sigma(\|F_o\|)$	7940	1661	1477	5477
**completeness (%)** [a]	99.4	98.4	98.5	99.9
**no. of variables**	751	130	136	439
R_1**(obs),** wR_2**(all)** [b]	0.0692, 0.1890	0.0421, 0.1151	0.0643, 0.1898	0.0385, 0.0918

[a] Completeness to 0.84 Å resolution. [b] $R_1 = \Sigma||F_o| - |F_c||/\Sigma|F_o|$; $wR_2 = \{\Sigma[w(F_o^2 - F_c^2)^2] / \Sigma[w(F_o^2)^2]\}^{1/2}$; $w^{-1} = \sigma^2(F_o^2) + (aP)^2 + bP$. [c] There are two crystallographically independent molecules.

Table S5. …part 2

data	(+)-**13**	**14**
**formula**	$C_{21}H_{17}BrO_2$	$C_8H_5ClN_2O$
**solvent**	—	—
**formula weight**	381.26	180.59
**colour, habit**	colourless blocks	colourless needles
**temperature / K**	173	173
**crystal system**	monoclinic	monoclinic
**space group**	*I*2 (no. 5)	$P2_1/c$ (no. 14)
a **/ Å**	14.0980(3)	3.7558(4)
b **/ Å**	7.50617(16)	13.4444(7)
c **/ Å**	16.0950(3)	14.5531(10)
**α / deg**	90	90
**β / deg**	92.4017(17)	91.564(7)
**γ / deg**	90	90
V **/ Å³**	1701.71(6)	734.58(9)
Z	4	4
D_c **/ g cm⁻³**	1.488	1.633
**radiation used**	Cu-Kα	Cu-Kα
**μ / mm⁻¹**	3.363	4.145
**no. of unique reflns**		
**measured (**R_{int}**)**	3205 (0.0224)	1445 (0.0520)
**obs,** $\lvert F_o \rvert > 4\sigma(\lvert F_o \rvert)$	3071	1133
**completeness (%)** [a]	99.9	99.9
**no. of variables**	218	113
R_1**(obs),** wR_2**(all)** [b]	0.0342, 0.0748	0.0620, 0.1800

Table S5 provides a summary of the crystallographic data for the structures of **8**, **9**, **11**, (±)-**13**, (+)-**13** and **14**. Data were collected using an Agilent Xcalibur PX Ultra A diffractometer, and the structures were solved and refined using the OLEX2,[3] SHELXTL[4] and SHELX-2013[5] program systems. The absolute structures of **8** and (+)-**13** were unambiguously determined by use of the Flack parameter [x = –0.03(3) and –0.032(11) respectively]. CCDC 2246905 to 2246910.

The X-ray crystal structure of 8

The N26–H and N50–H hydrogen atoms in the structure of **8** were located from ΔF maps and refined freely subject to an N–H distance constraint of 0.90 Å. The included solvent was found to be highly disordered, and the best approach to handling this diffuse electron density was found to be the SQUEEZE routine of

PLATON.[6] This suggested a total of 69 electrons per unit cell, equivalent to 34.5 electrons per molecule. Before the use of SQUEEZE the solvent clearly resembled methanol (CH_4O, 18 electrons), and 2 methanol molecules corresponds to 36 electrons, so this was used as the solvent present. As a result, the atom list for the asymmetric unit is low by $2(CH_4O) = C_2H_8O_2$ (and that for the unit cell low by $C_4H_{16}O_4$) compared to what is actually presumed to be present. The absolute structure of **8** was unambiguously determined by use of the Flack parameter [x = –0.03(3)].

The X-ray crystal structure of 11

The C3-based *t*-butyl group in the structure of **11** was found to be disordered. Two orientations were identified of *ca*. 67 and 33% occupancy, their geometries were optimised, the thermal parameters of adjacent atoms were restrained to be similar, and only the non-hydrogen atoms of the major occupancy orientation were refined anisotropically (those of the minor occupancy orientation were refined isotropically). The N1–H hydrogen atom was located from a ΔF map and refined freely subject to an N–H distance constraint of 0.90 Å.

The X-ray crystal structure of (±)-13

The structure of (±)-**13** was found to contain two crystallographically independent molecules [(±)-**13-A** and (±)-**13-B**] in the asymmetric unit. Interestingly the two independent molecules have different conformations, with molecule (±)-**13-A** having the naphthoate and bromine substituents in axial positions, whereas molecule (±)-**13-B** has them in equatorial positions. A single large residual electron density peak of *ca*. 2.26 $eÅ^{-3}$ near C8B was modelled as a low occupancy (*ca*. 6%) bromine atom of a second orientation of the whole of molecule (±)-**13-B** for which the other atoms did not possess sufficient electron density to be spotted above the background (this atom was refined isotropically).

The X-ray crystal structure of (+)-13

The structure of (+)-**13** reported here is the same as that already reported by Shi *et al*.[7] (CCDC refcode QOJQEN). The absolute structure of (+)-**13** was unambiguously determined by use of the Flack parameter [x = –0.032(11)].

The X-ray crystal structure of 14

The N3–H hydrogen atom in the structure of **14** was located from a Δ*F* map and refined freely subject to an N–H distance constraint of 0.90 Å.

Figures

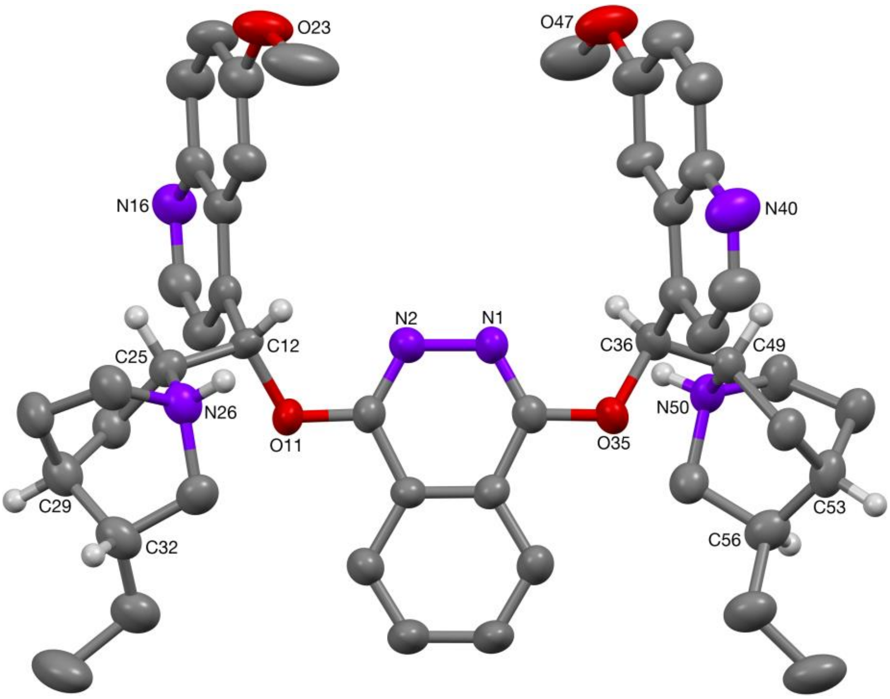

Figure S5. The crystal structure of **8** (50% probability ellipsoids).

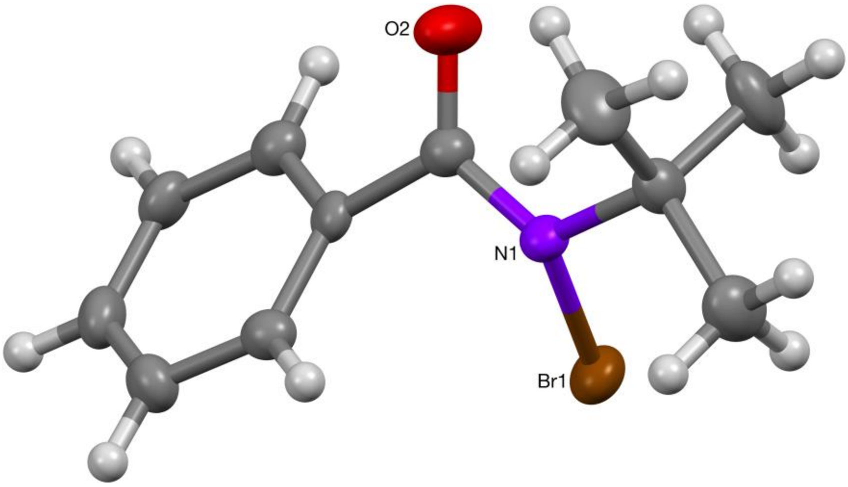

Figure S6. The crystal structure of **9** (50% probability ellipsoids).

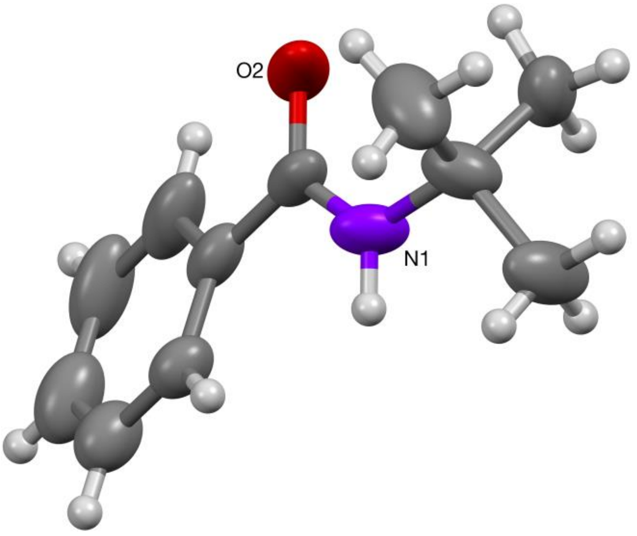

Figure S7. The crystal structure of **11** (50% probability ellipsoids).

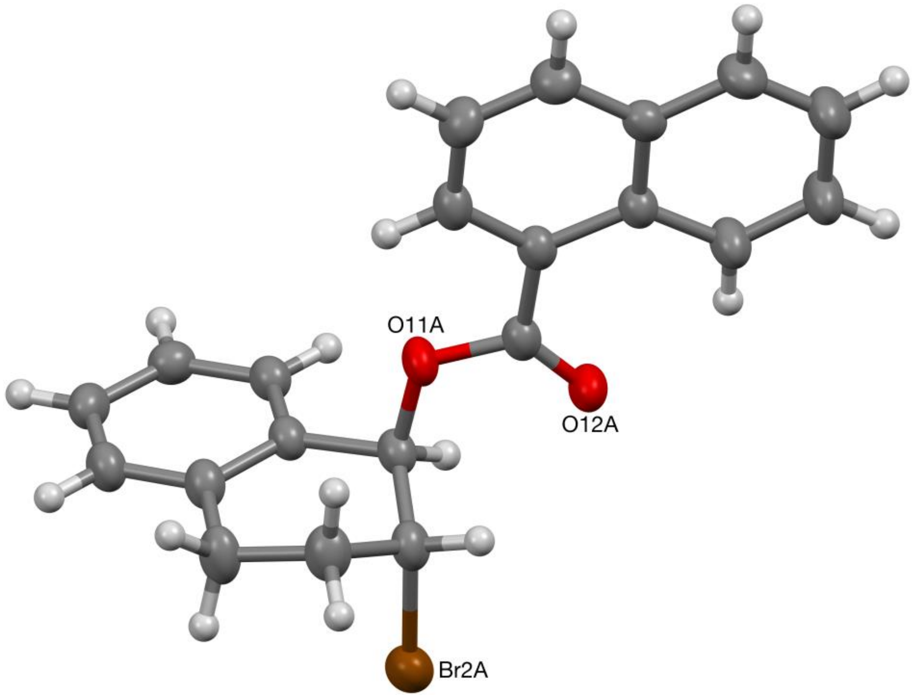

Figure S8. The structure of (±)-**13-A**, one of the two independent molecules present in the crystal of (±)-**13** (50% probability ellipsoids).

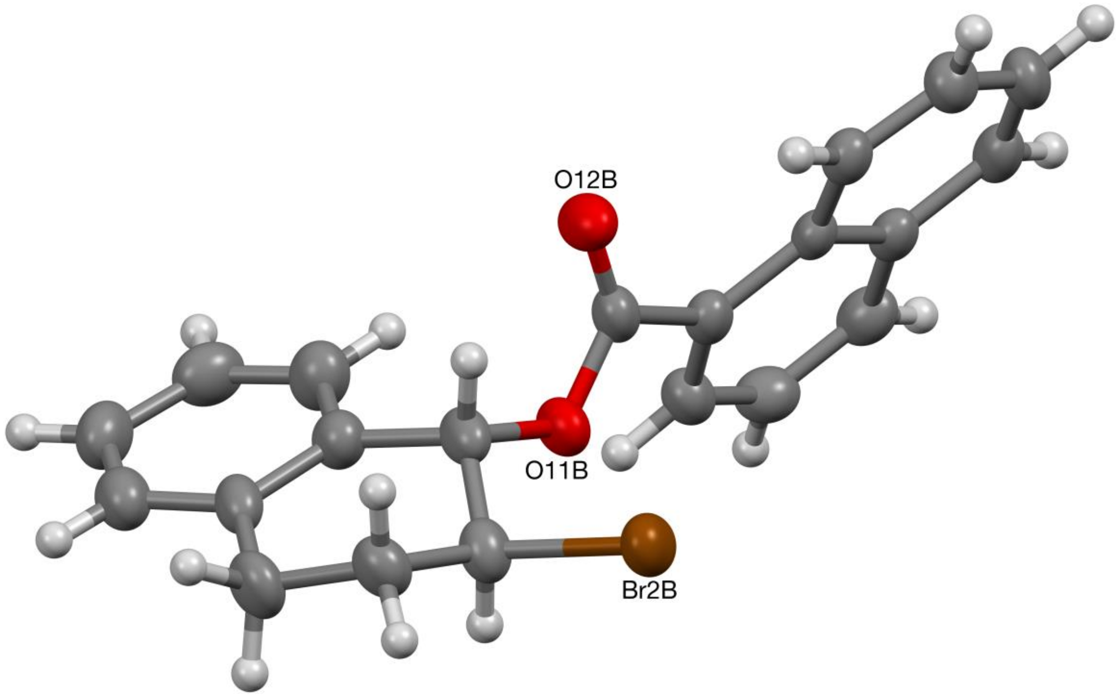

Figure S9. The structure of (±)-**13-B**, one of the two independent molecules present in the crystal of (±)-**13** (50% probability ellipsoids).

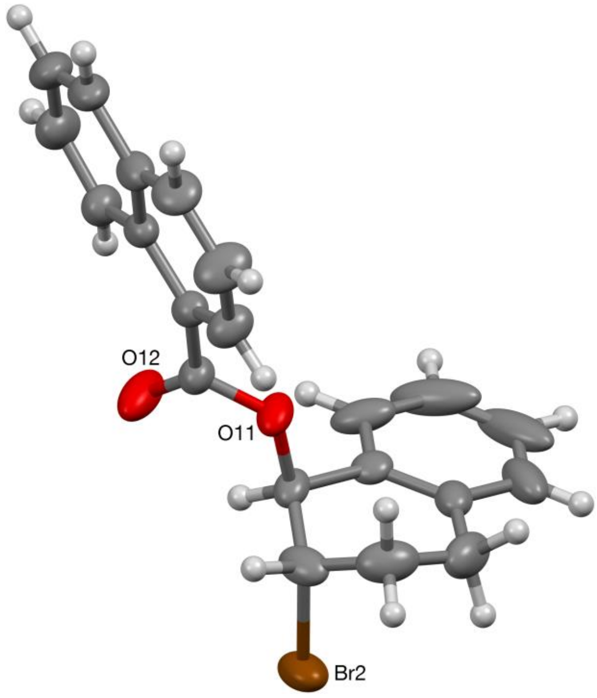

Figure S10. The crystal structure of (+)-**13** (50% probability ellipsoids).

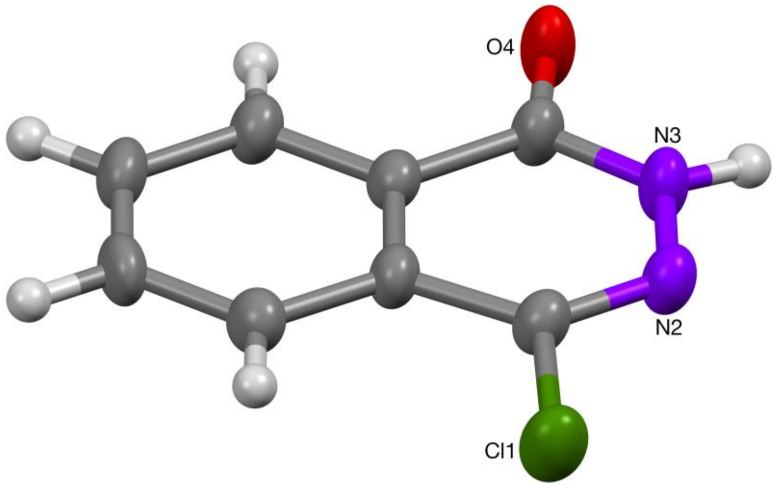

Figure S11. The crystal structure of **14** (50% probability ellipsoids).

7. HPLC Calibration Curves

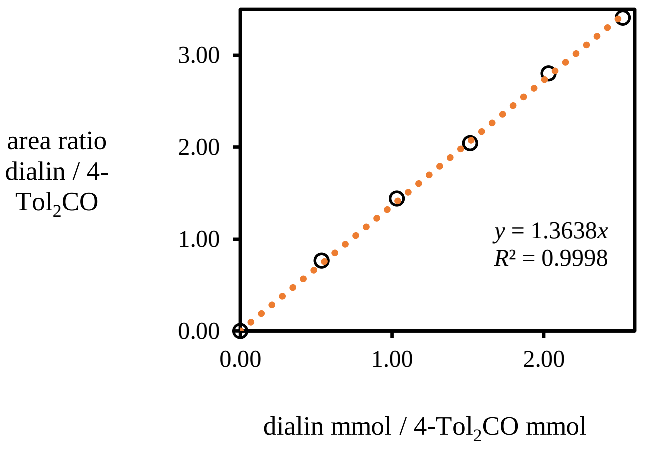

Figure S12. HPLC calibration of dialin (**2**) and 4-Tol_2CO (**7**) at a detection wavelength of 220 nm.

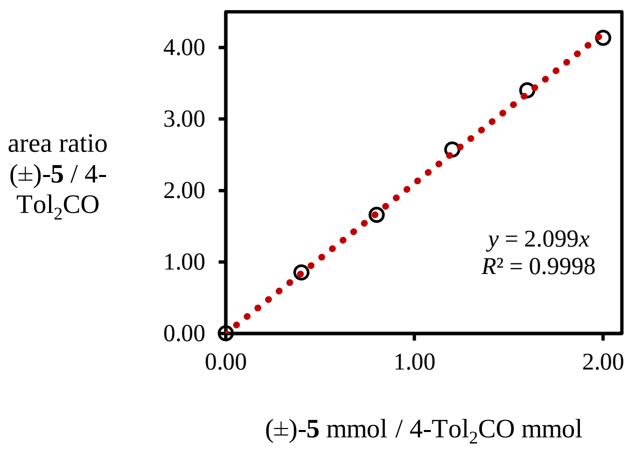

Figure S13. HPLC calibration of (±)-**5** and 4-Tol_2CO (**7**) at a detection wavelength of 230 nm.

8. Copies of ^{1}H and ^{13}C{^{1}H} NMR Spectra

^{1}H NMR spectrum of PhCONHBr (**3**) (400 MHz, acetone-d_6)

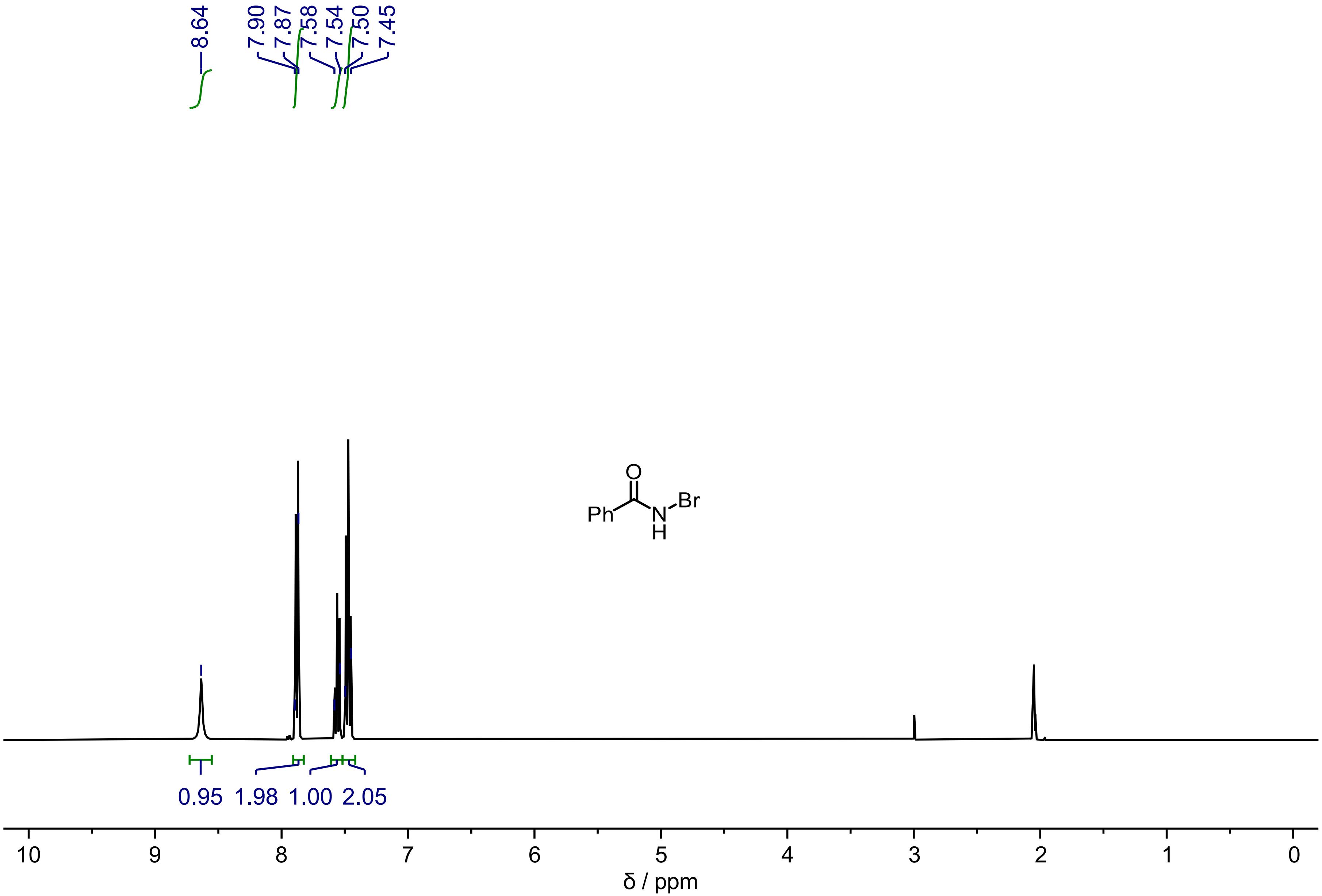

$^{13}C\{^{1}H\}$ NMR spectrum of PhCONHBr (**3**) (101 MHz, acetone-d_6)

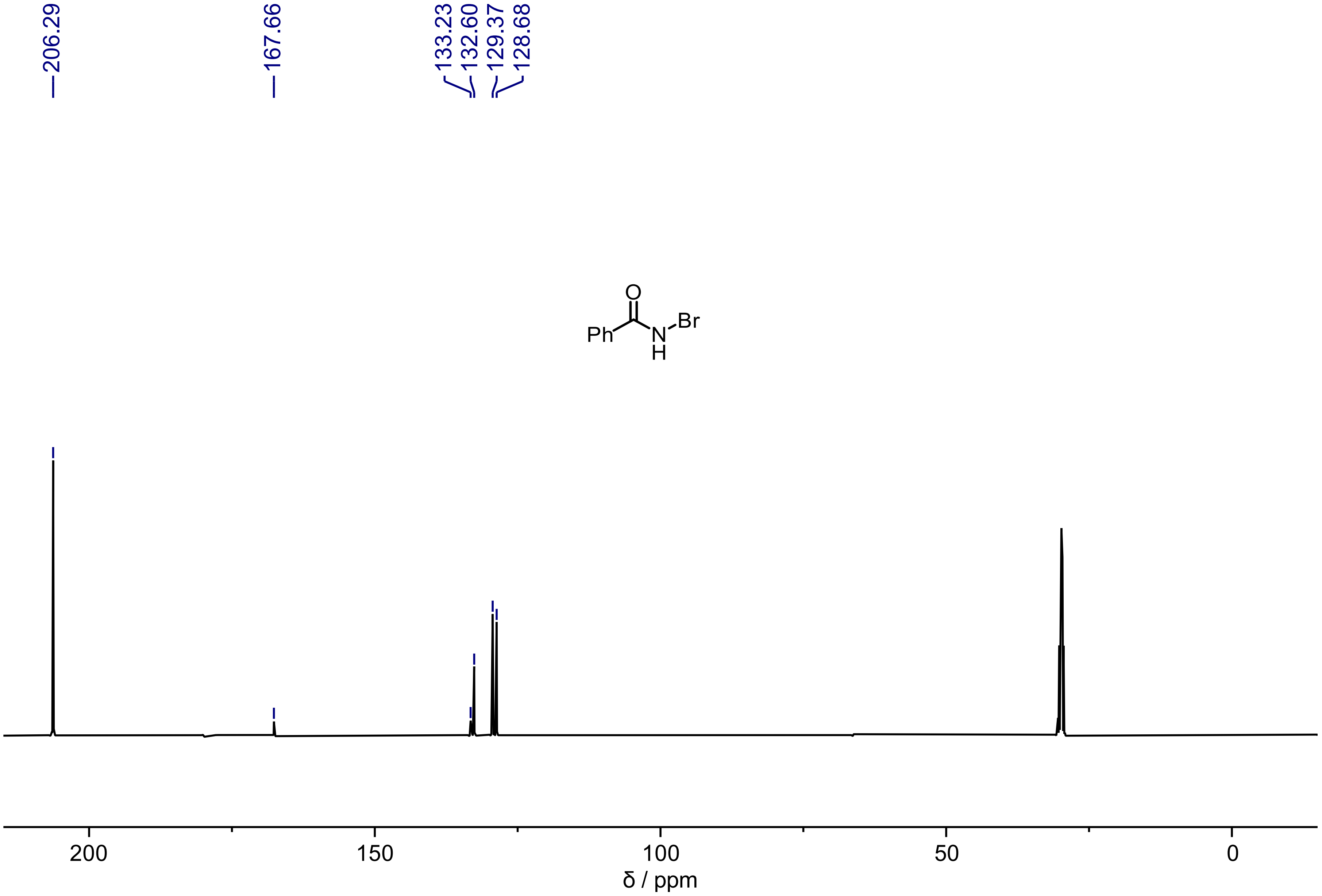

^{1}H NMR spectrum of (+)-(1*S*,2*S*)-2-bromo-1,2,3,4-tetrahydronaphthalen-1-yl benzoate (**5**) (400 MHz, $CDCl_3$)

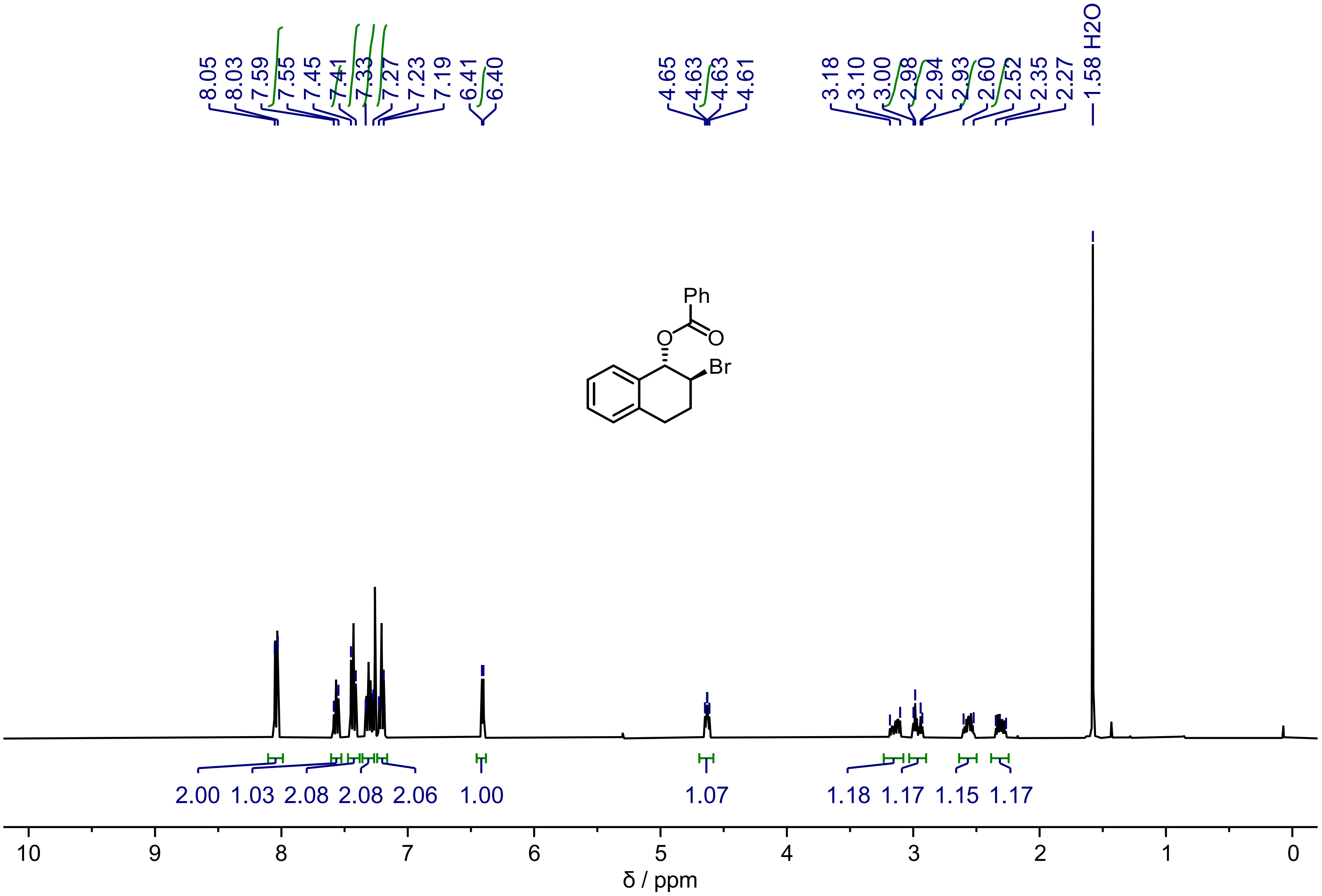

$^{13}C\{^{1}H\}$ NMR spectrum of (+)-(1*S*,2*S*)-2-bromo-1,2,3,4-tetrahydronaphthalen-1-yl benzoate (**5**) (101 MHz, $CDCl_3$)

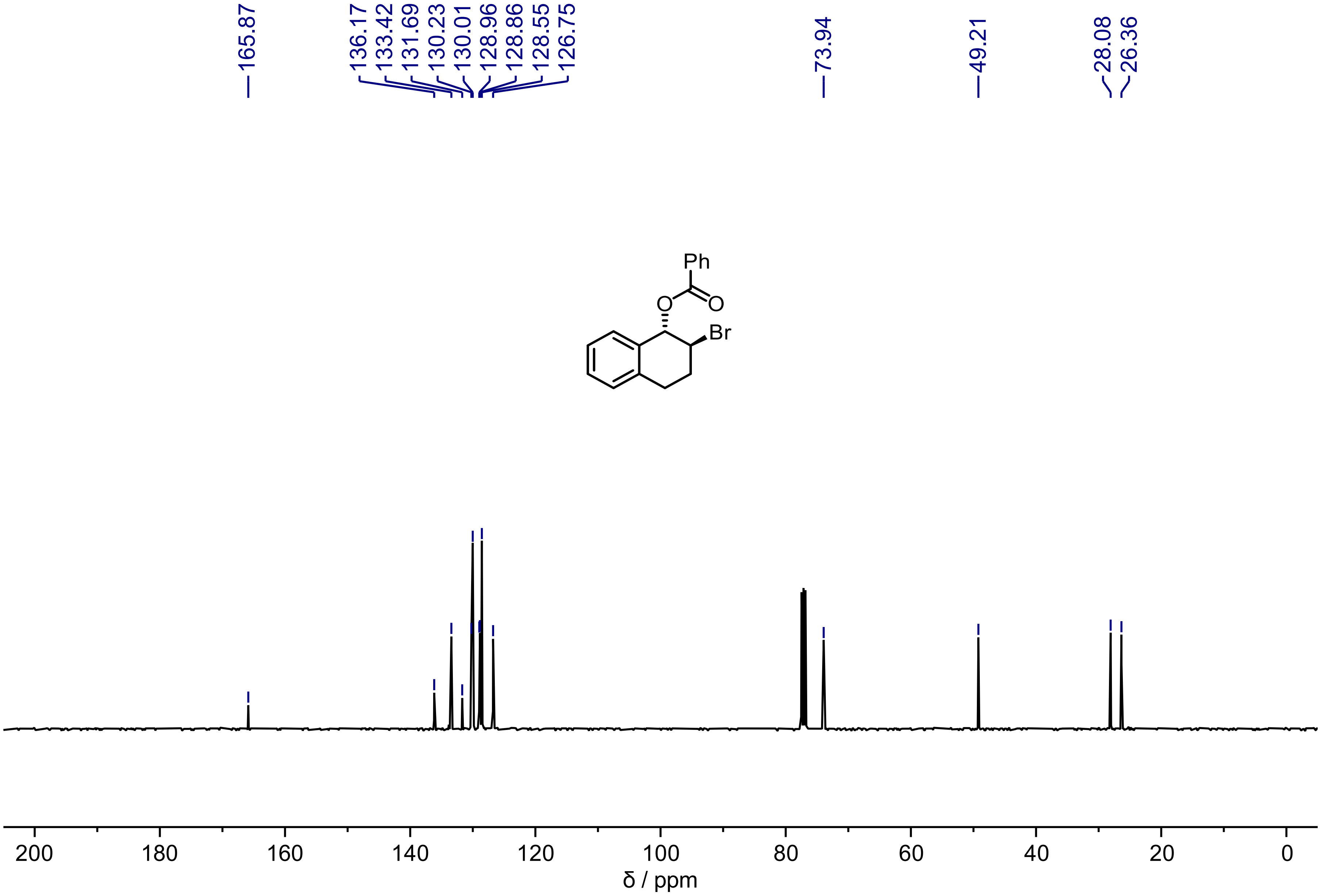

^{1}H NMR spectrum of **4**•(Saccharin)$_2$ (**8**) (400 MHz, $CDCl_3$)

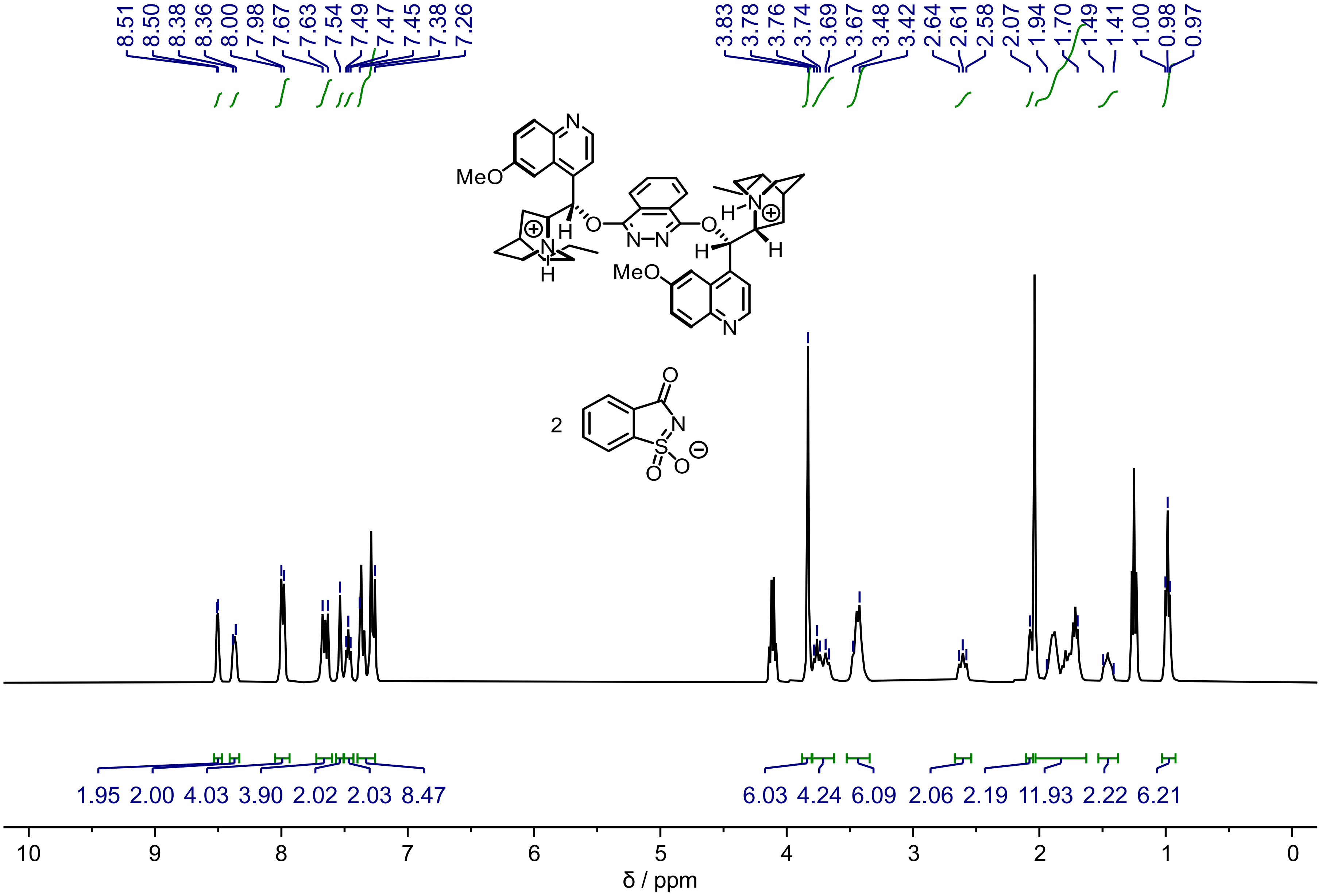

$^{13}C\{^{1}H\}$ NMR spectrum of **4**•(Saccharin)$_2$ (**8**) (101 MHz, $CDCl_3$)

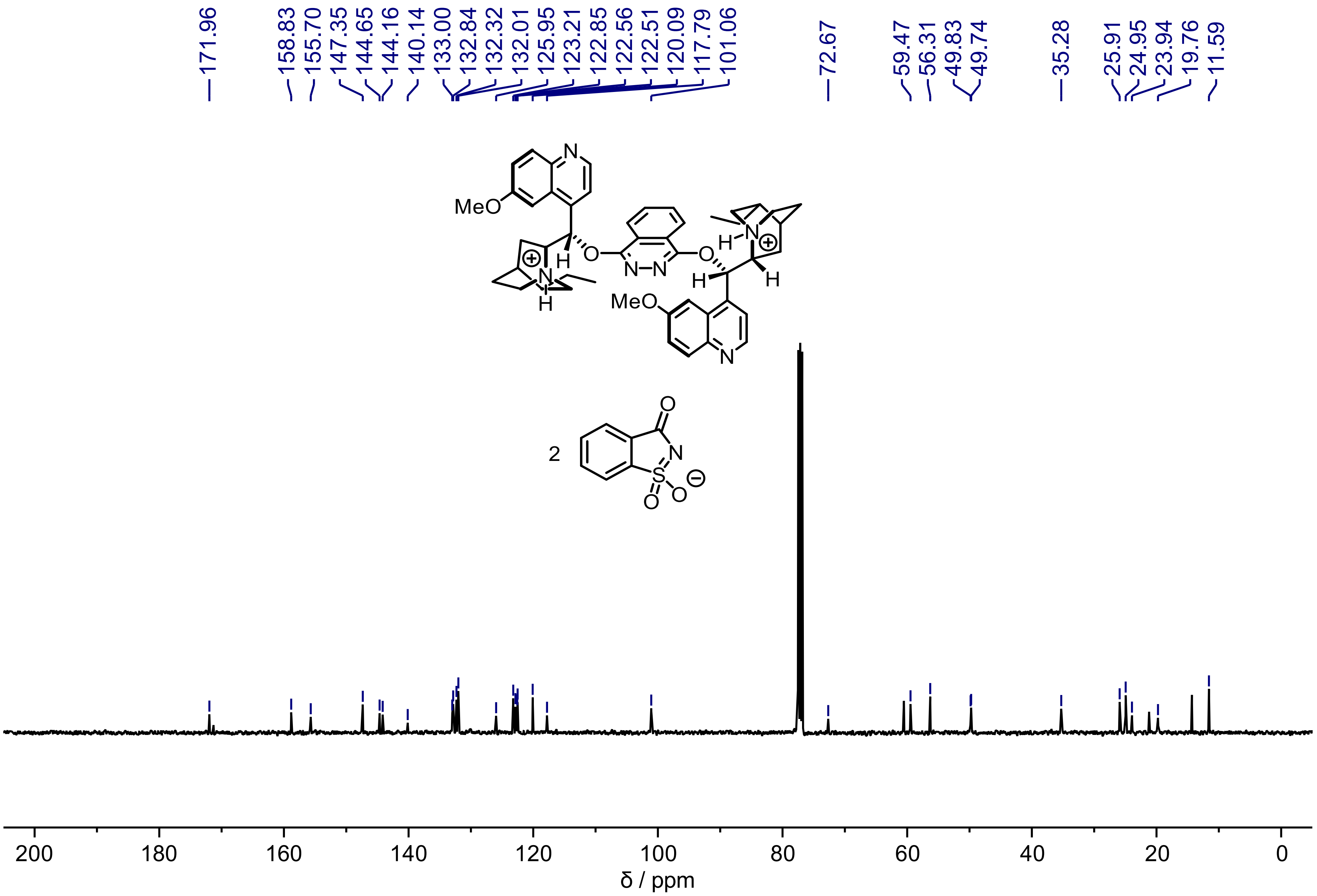

^{1}H NMR spectrum of PhCONBr(*t*-Bu) (**9**) (400 MHz, $CDCl_3$)

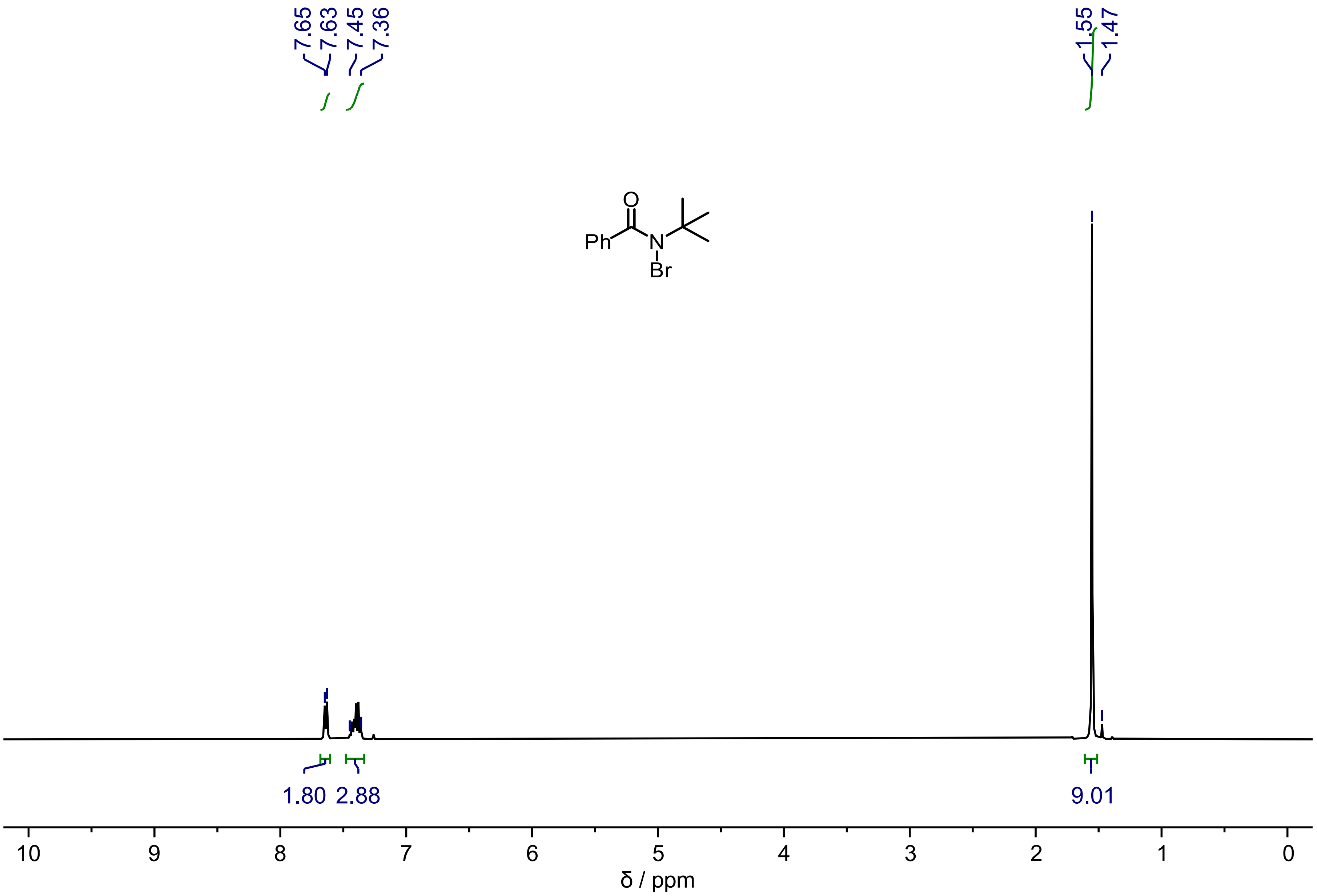

$^{13}C\{^{1}H\}$ NMR spectrum of PhCONBr(*t*-Bu) (**9**) (101 MHz, $CDCl_3$)

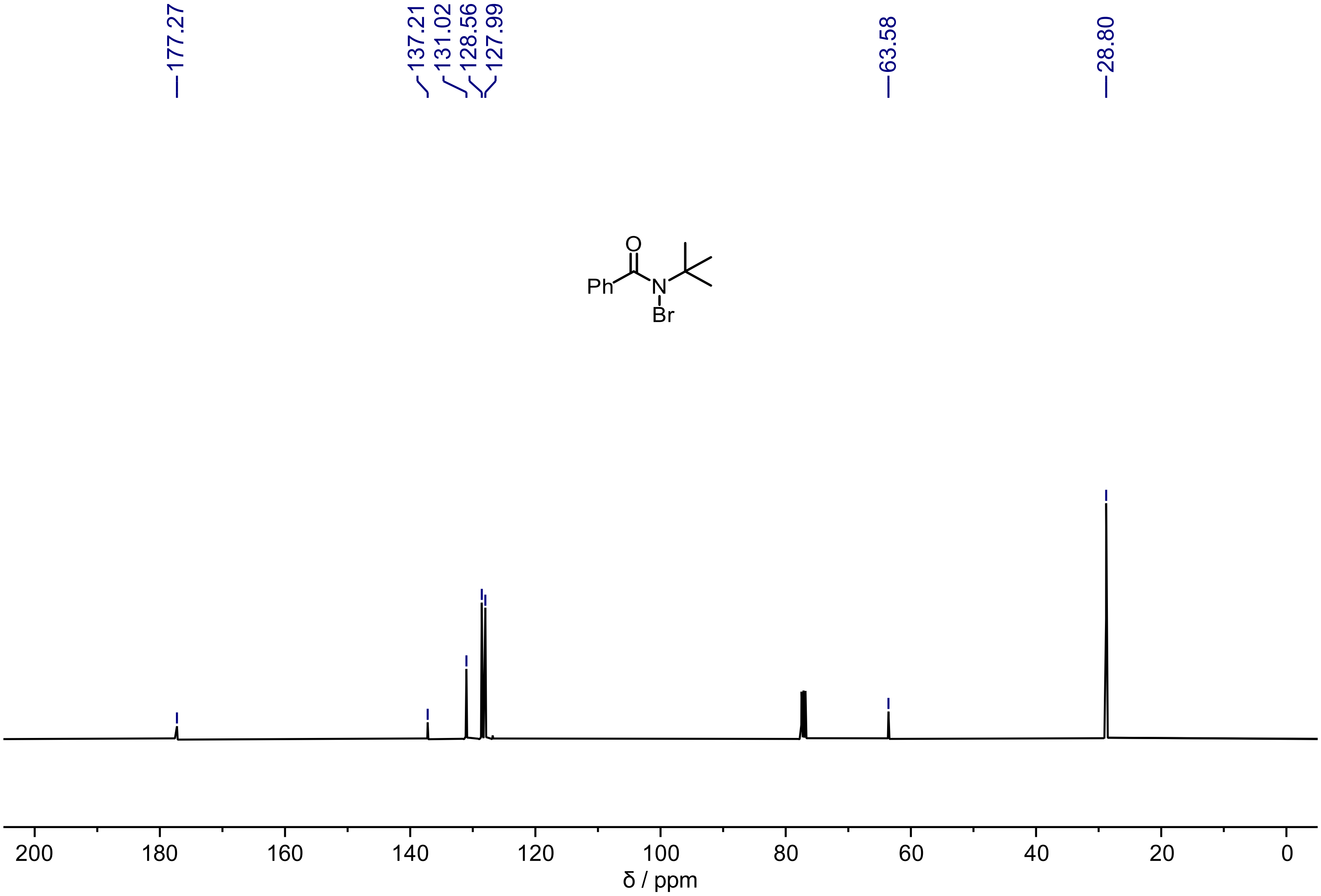

^{1}H NMR spectrum of PhCONBrMe (**10**) (400 MHz, $CDCl_3$)

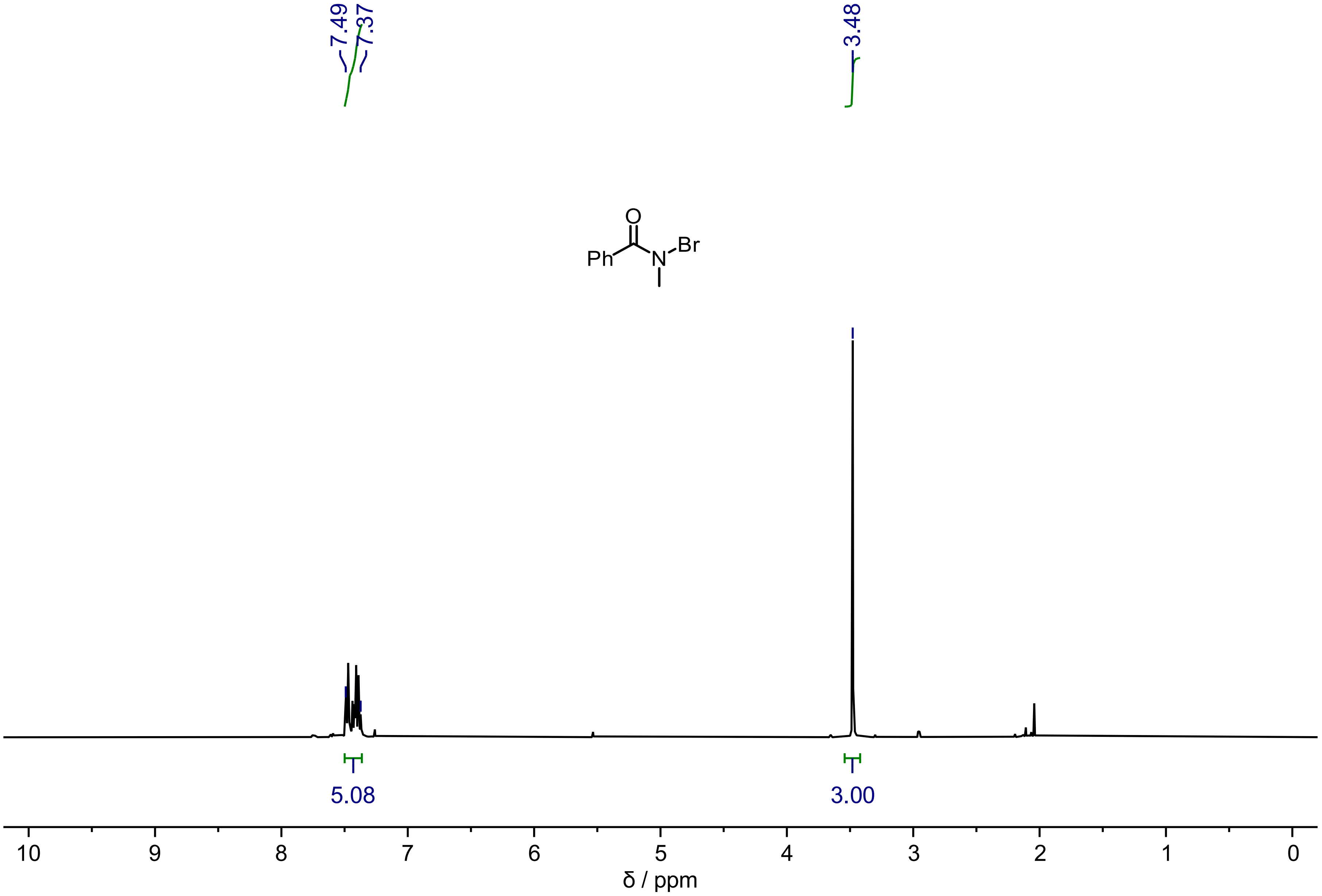

$^{13}C\{^{1}H\}$ NMR spectrum of PhCONBrMe (**10**) (101 MHz, $CDCl_3$)

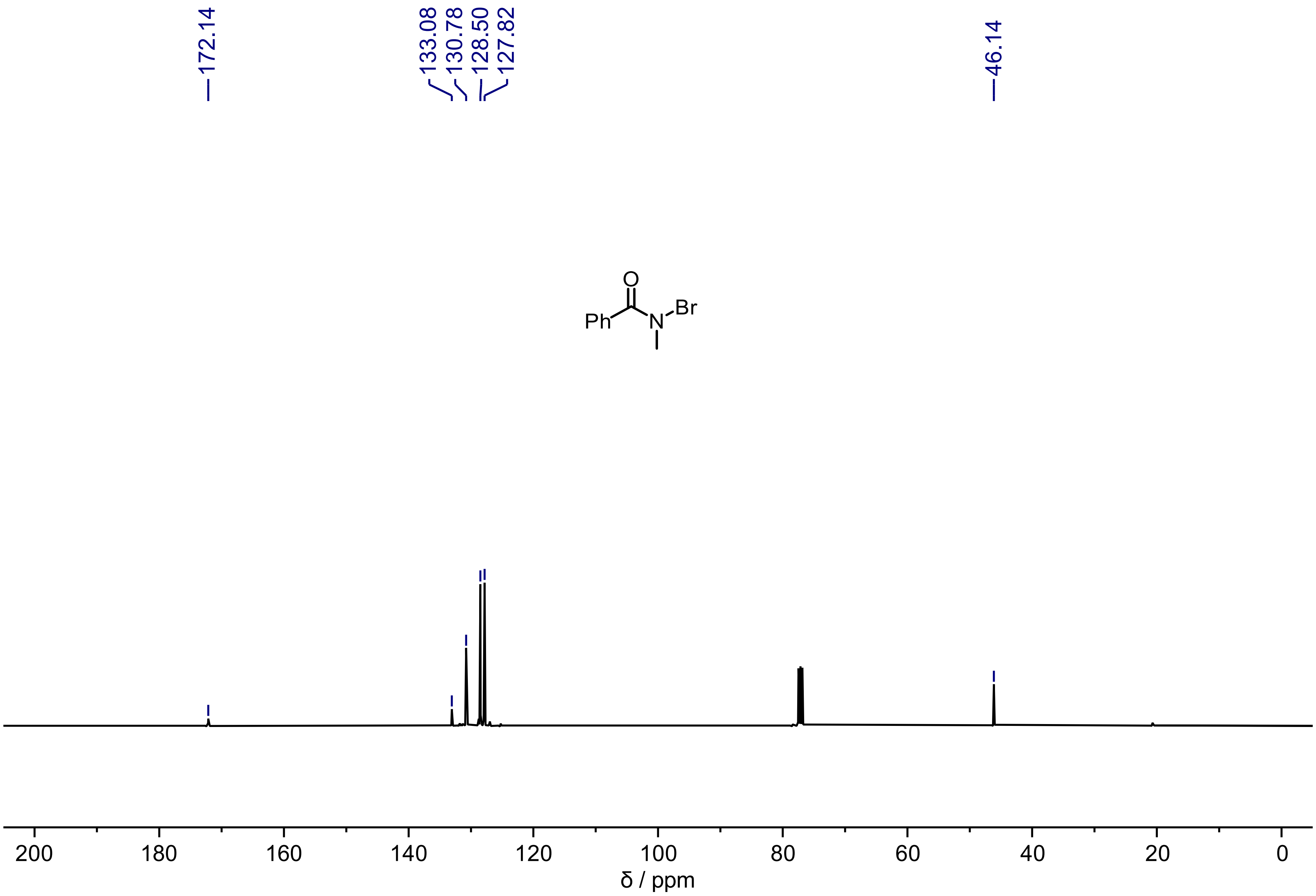

^{1}H NMR spectrum of (+)-(1*S*,2*S*)-2-bromo-1,2,3,4-tetrahydronaphthalen-1-yl 1-naphthoate (**13**) (400 MHz, $CDCl_3$)

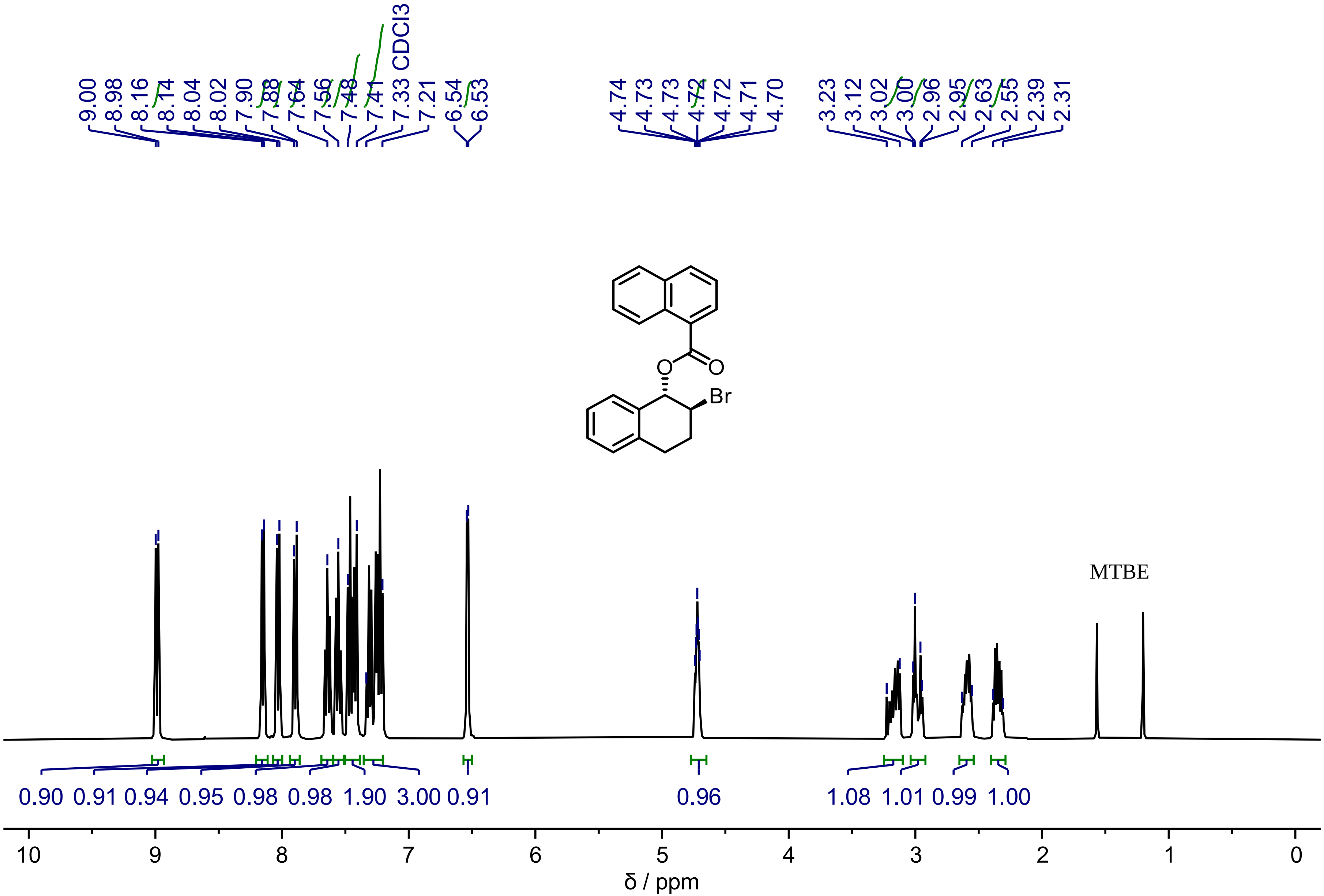

$^{13}C\{^1H\}$ NMR spectrum of (+)-(1*S*,2*S*)-2-bromo-1,2,3,4-tetrahydronaphthalen-1-yl 1-naphthoate (**13**) (101 MHz, $CDCl_3$)

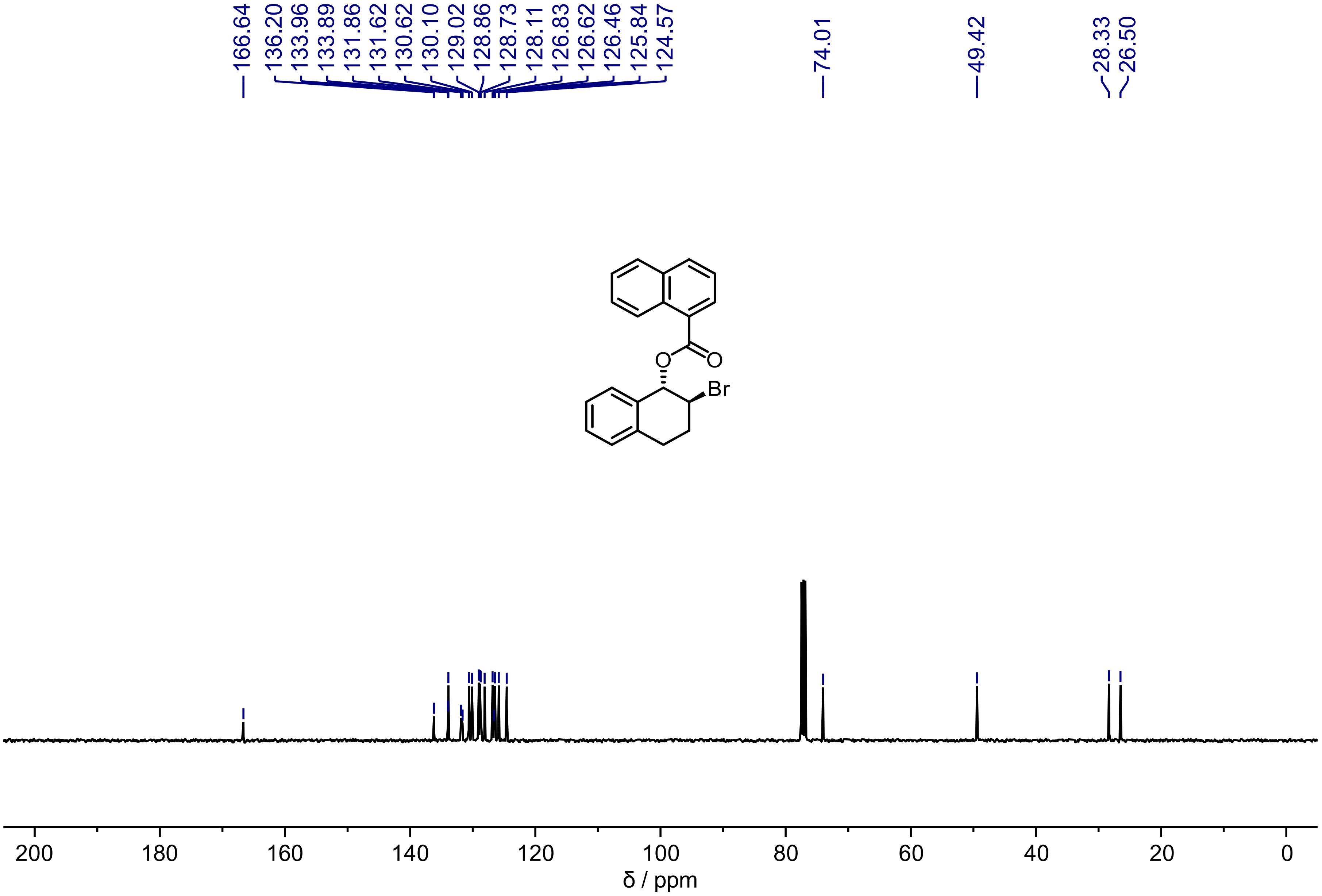

^{1}H NMR spectrum of (−)-(1*S*,2*S*)-2-bromo-1,2,3,4-tetrahydronaphthalen-1-ol (**15**) (400 MHz, $CDCl_3$)

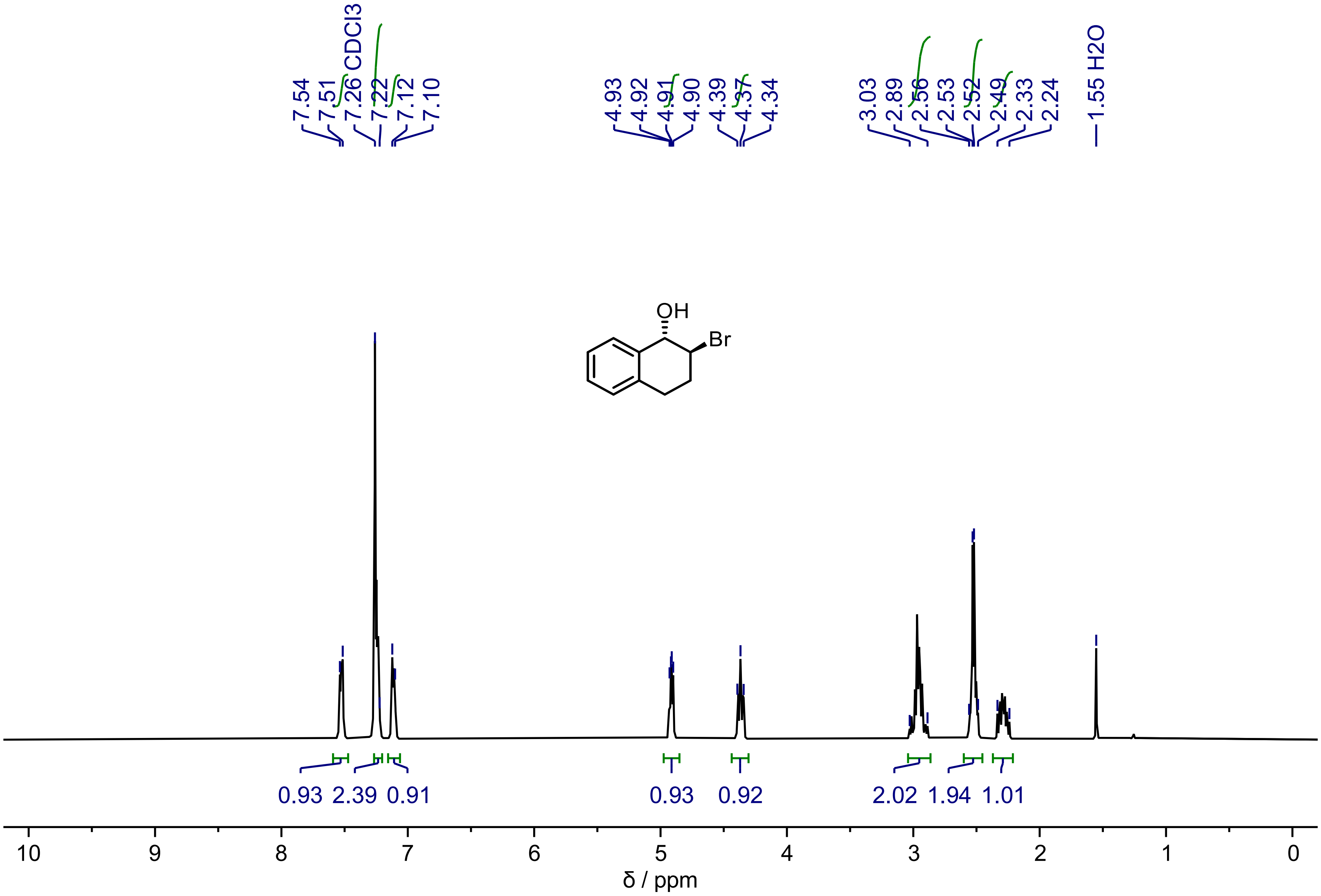

$^{13}C\{^{1}H\}$ NMR spectrum of (−)-(1*S*,2*S*)-2-bromo-1,2,3,4-tetrahydronaphthalen-1-ol (**15**) (101 MHz, $CDCl_3$)

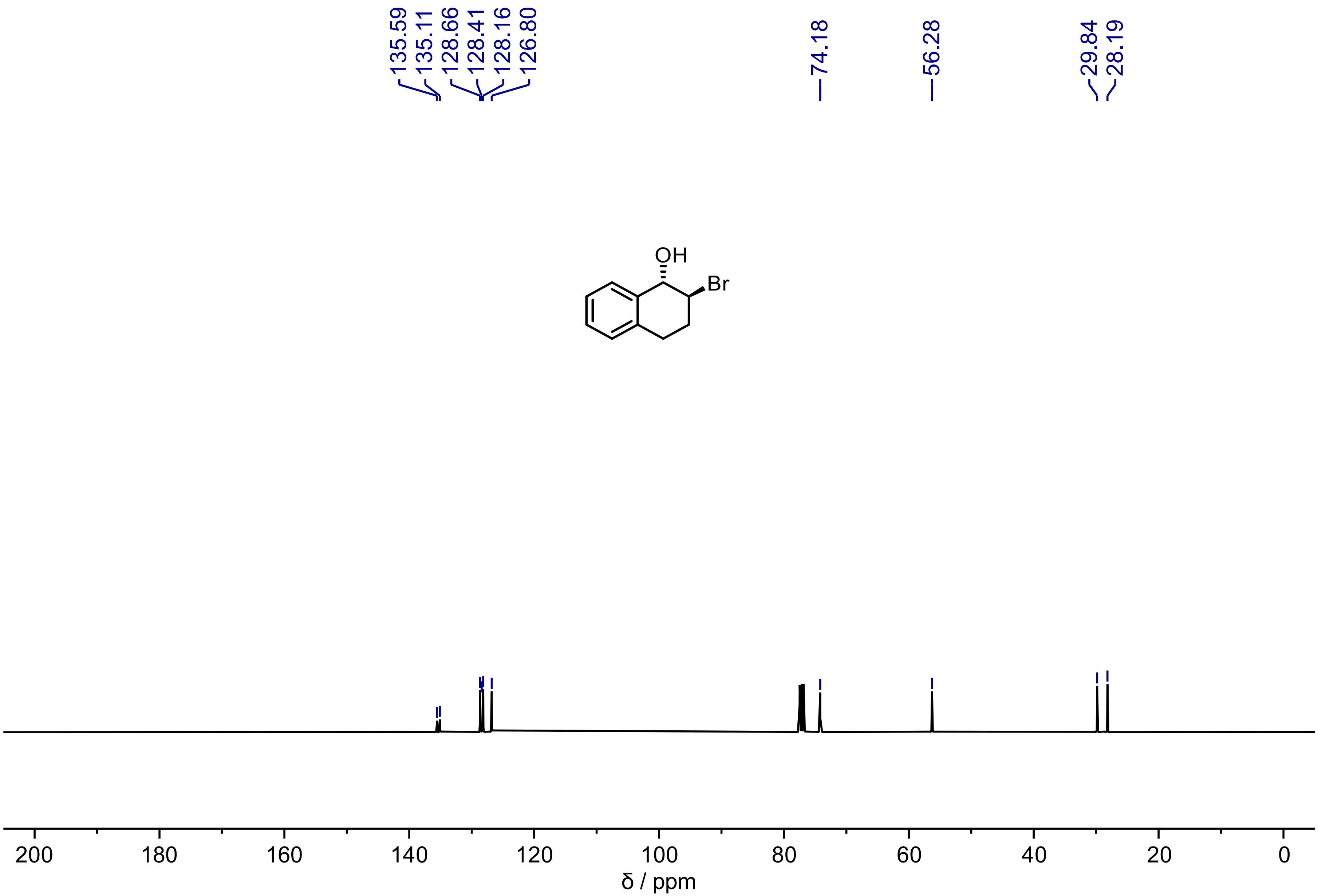

9. Representative HPLC Chromatograms

Representative HPLC chromatogram of (+)-(1*S*,2*S*)-2-bromo-1,2,3,4-tetrahydronaphthalen-1-yl benzoate [(+)-**5**] and comparison with (−)-(1*R*,2*R*)-2-bromo-1,2,3,4-tetrahydronaphthalen-1-yl benzoate [(−)-**5**]

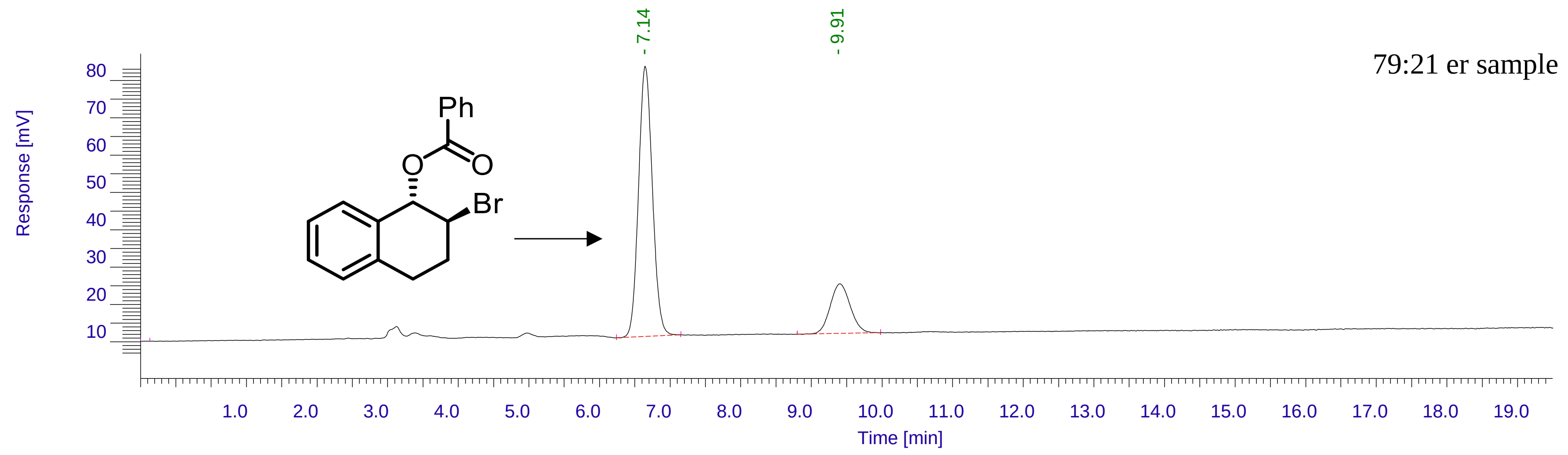

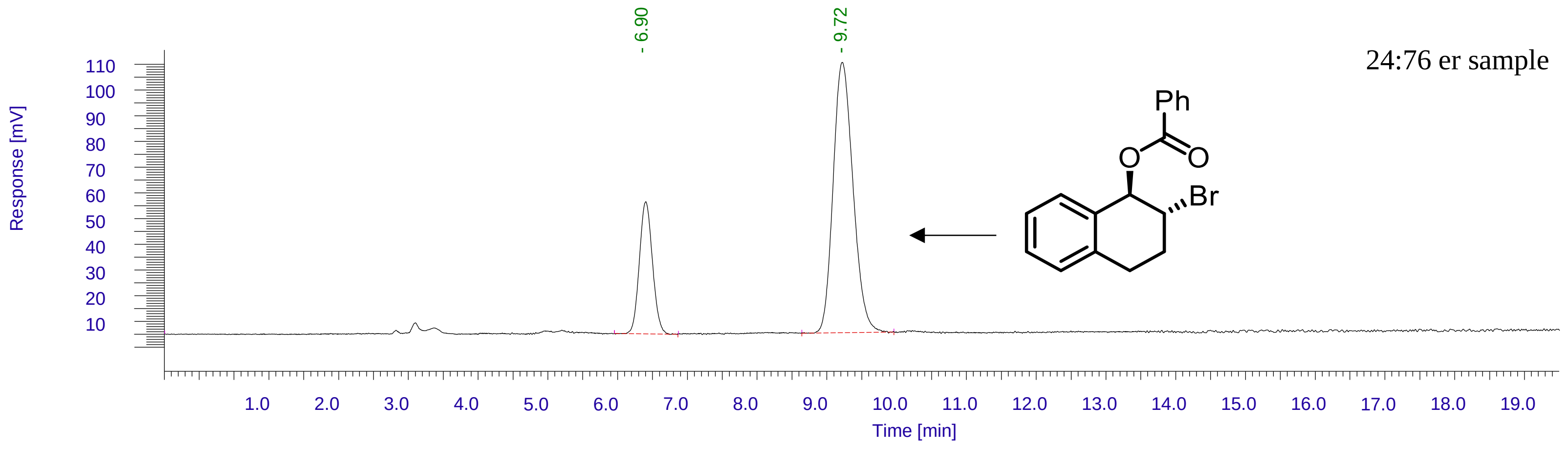

Conditions: 10 µm CHIRALPAK-AD, 99% *n*-hexane, 1% isopropanol, 1.0 mL/min, RT, 230 nm, 5 µL injection

Representative HPLC chromatograms of (−)-(1*S*,2*S*)-2-bromo-1,2,3,4-tetrahydronaphthalen-1-ol (**15**) from DIBAL-H reduction of bromoester **13** and comparison with scalemic (−)-(1*S*,2*S*)-2-bromo-1,2,3,4-tetrahydronaphthalen-1-ol (**15**)

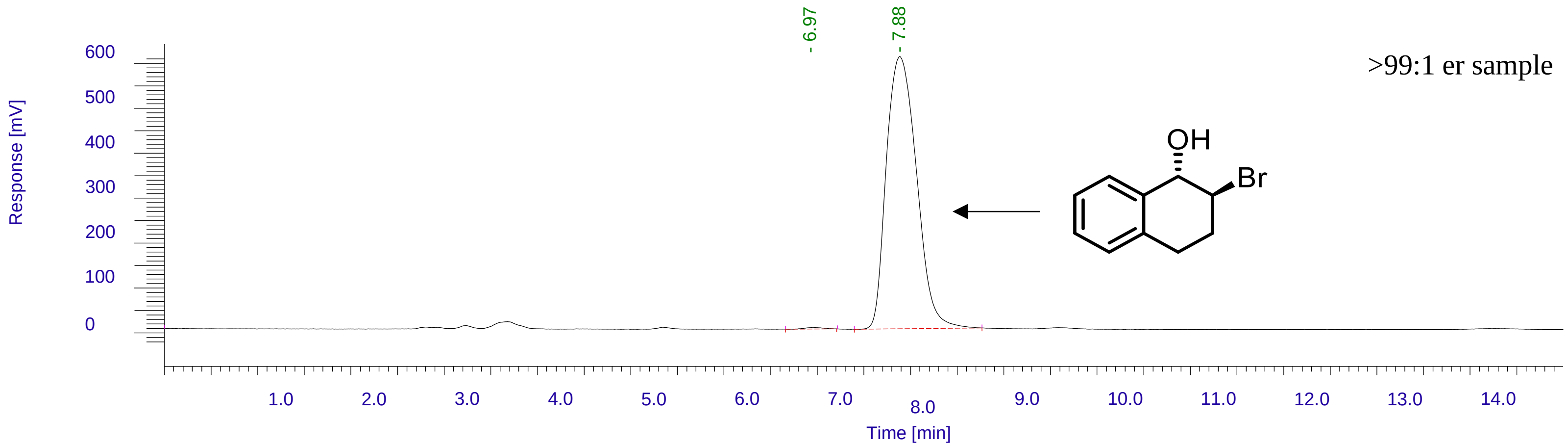

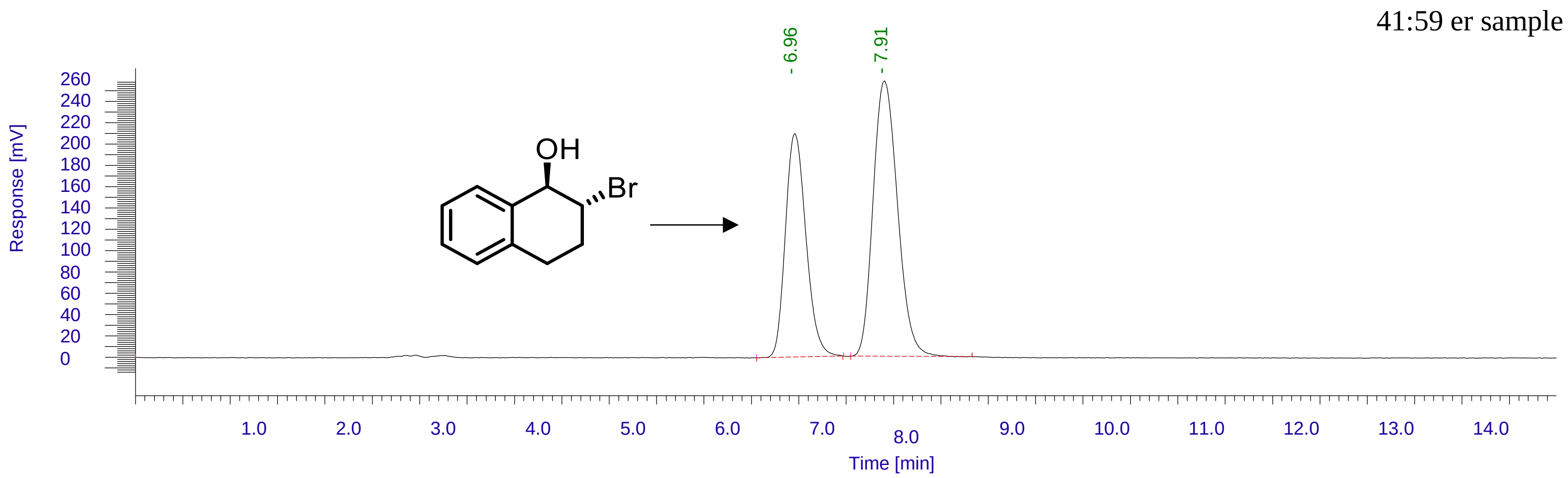

Conditions: 10 µm CHIRALPAK-AD, 90% *n*-hexane, 10% isopropanol, 1.0 mL/min, RT, 220 nm, 5 µL injection.

10. References

1. Schmidt, V. A.; Quinn, R. K.; Brusoe, A. T.; Alexanian, E. J. Site-Selective Aliphatic C–H Bromination Using *N*-Bromoamides and Visible Light. *J. Am. Chem. Soc.* **2014**, *136*, 14389−14392. DOI: 10.1021/ja508469u.
2. Yousefi, R.; Sarkar, A.; Ashtekar, K. D.; Whitehead, D. C.; Kakeshpour, T.; Holmes, D.; Reed, P.; Jackson, J. E.; Borhan, B. Mechanistic Insights into the Origin of Stereoselectivity in an Asymmetric Chlorolactonization Catalyzed by $(DHQD)_2PHAL$. *J. Am. Chem. Soc.* **2020**, *142*, 7179−7189. DOI: 10.1021/jacs.0c01830.
3. Dolomanov, O. V.; Bourhis, L. J.; Howard, J. A. K.; Puschmann, H. *OLEX2*: a complete structure solution, refinement and analysis program. *J. App. Cryst.* **2009**, *42*, 339–341. DOI: 10.1107/S0021889808042726.
4. SHELXTL v5.1, Bruker AXS, Madison, WI, 1998.
5. Sheldrick, G. M. Crystal structure refinement with *SHELXL*. *Acta Cryst.* **2015**, *71*, 3–8. DOI: 10.1107/S2053229614024218.
6. (a) Spek, A. L. Single-crystal structure validation with the program *PLATON*. *J. App. Cryst.* **2003**, *36*, 7–13. DOI: 10.1107/S0021889802022112; (b) Spek, A. L. *PLATON* SQUEEZE: a tool for the calculation of the disordered solvent contribution to the calculated structure factors. *Acta Cryst.* **2015**, *71*, 9–18. DOI: 10.1107/s2053229614024929.
7. Li, L.; Su, C.; Liu, X.; Tian, H.; Shi, Y. Catalytic Asymmetric Intermolecular Bromoesterification of Unfunctionalized Olefins. *Org. Lett.* **2014**, *16*, 3728−3731. DOI: 10.1021/ol501542r.
